# Bone Marrow Macrophage STING Signaling is a Direct Nexus Linking Marrow Adipose Expansion to NAD^+^ Depletion and Osteogenic Decline in Skeletal Aging

**DOI:** 10.1002/advs.77571

**Published:** 2026-10-15

**Authors:** Zhichao Li, Kuanhui Gao, Mengjie Wang, Songlin Liang, Dandan Li, Guoqing Tan, Haipeng Xue, Yujie Liu, Peng Zhang, Zhanwang Xu, Wenxiang Cheng, Yongjun Wang, Nianhu Li

**Affiliations:** ^1^ Center For Translational Medicine Research and Development Shenzhen Institute of Advanced Technology Chinese Academy of Sciences Shenzhen China; ^2^ First College of Clinical Medicine Shandong University of Traditional Chinese Medicine Jinan China; ^3^ Department of Orthopedics Affiliated Hospital of Shandong University of Traditional Chinese Medicine Jinan China; ^4^ College of Integrated Traditional Chinese and Western Medicine Hebei University of Chinese Medicine Shijiazhuang China; ^5^ Faculty of Biomedical Engineering Shenzhen University of Advanced Technology Shenzhen China; ^6^ Key Laboratory of Biomedical Imaging Science and System Chinese Academy of Sciences Shenzhen China; ^7^ Longhua Hospital Shanghai University of Traditional Chinese Medicine Shanghai China; ^8^ Spine Institute Shanghai University of Traditional Chinese Medicine Shanghai China; ^9^ Key Laboratory of Theory and Therapy of Muscles and Bones Ministry of Education (Shanghai University of Traditional Chinese Medicine) Shanghai China

**Keywords:** bone marrow macrophage, cGAS‐STING, dipeptidyl peptidase 4, NAD^+^, osteoporosis

## Abstract

Reversing the destructive remodeling of the bone marrow microenvironment during aging remains a major challenge. Here, we reveal an age‐dependent dual role of bone marrow macrophage (BMM) STING signaling during skeletal aging. While BMM STING deficiency is detrimental during early bone development and maturation, it paradoxically confers protection against age‐related bone loss from middle age onward. Mechanistically, accumulating bone marrow adipocytes secrete substantial soluble dipeptidyl peptidase 4 (sDPP4). This sDPP4 functions as an extracellular ligand for Toll‐like receptor 4 on BMMs, thereby collaborating with endogenous mitochondrial DNA to activate STING signaling. This STING activation concurrently induces a CD38‐dependent salvage synthesis pathway. Importantly, this STING‐driven upregulation of CD38 not only amplifies bone marrow inflammation but also, through its ecto‐nicotinamide adenine dinucleotide (NAD^+^) activity, depletes the precursor nicotinamide mononucleotide (NMN) required for NAD^+^ salvage synthesis, thereby impairing NMN availability and osteogenic capacity in neighboring bone marrow mesenchymal stem cells. Finally, therapeutic intervention combining STING pharmacological inhibition with NMN supplementation effectively attenuated bone loss in preclinical models. Thus, we propose BMM STING signaling as a direct nexus connecting marrow adipose accumulation, innate immune inflammation, and diminished bone formation, and emphasize its targeting potential for managing the aged bone marrow microenvironment.

## Graphical abstract

This study reveals that during skeletal aging, bone marrow adipocyte‐derived sDPP4 primes STING signaling in macrophages. In combination with mitochondrial DNA, this signaling activates CD38 and depletes local NAD^+^/NMN, thereby impairing osteogenesis. Pharmacological STING inhibition, combined with NMN supplementation, attenuates bone loss, highlighting this pathway as a potential therapeutic target.

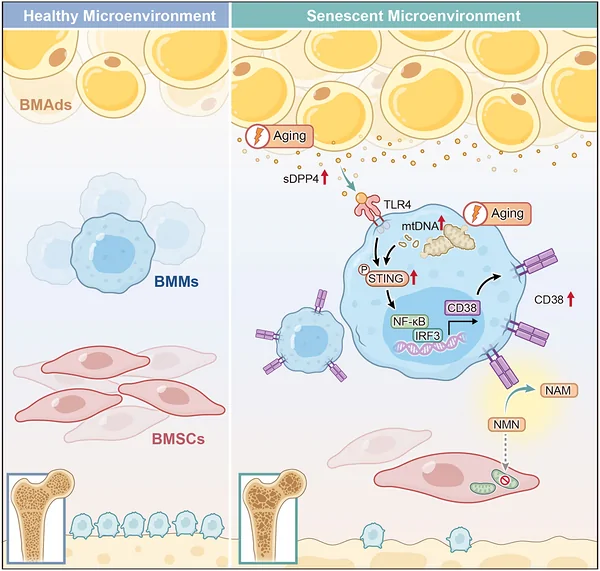

## 1 Introduction

The dynamic equilibrium of bone remodeling is orchestrated by the coordinated activities of diverse cellular lineages residing within the bone marrow microenvironment. These include cells derived from the mesenchymal lineage, such as bone marrow mesenchymal stem cells (BMSCs) and their descendants at various differentiation stages before reaching terminal states like osteoblasts (Obs), osteocytes, and bone marrow adipocytes (BMAds), as well as cells of hematopoietic origin, including bone marrow macrophages (BMMs) and osteoclasts (Ocs) [1, 2]. However, aging disrupts this balance by altering the quantity and function of these cellular constituents, progressively driving the bone marrow niche toward a pathology‐prone state that favors bone loss. Among the most prominent aging‐associated pathological alterations is the expansion of bone marrow adipose tissue (BMAT), which arises from the accumulation of BMAds [3]. Importantly, even after stringent adjustment for confounding variables such as age, sex, race, ethnicity, menopausal status, and body composition, BMAT expansion remains strongly correlated with hallmarks of osteoporosis [4]. Studies report that the accumulation of BMAds not only occupies bone marrow niches, thereby restricting the growth of other cells, but also rapidly acquires a senescence‐associated secretory phenotype (SASP) under certain stimuli, for example, glucocorticoid treatment, thereby propagating secondary senescence to the surrounding bone [5]. Similarly, aged bone marrow exhibits accumulation of pro‐inflammatory and senescent immune cells, notably macrophages and neutrophils, which further contribute to bone loss [6]. Collectively, these observations suggest that synergistic interactions may arise among pathologically altered bone marrow cell populations during advanced aging. In contrast to well‐characterized SASP factors from other bone marrow cells, such as proliferating cell nuclear antigen clamp‐associated factor derived from BMMs [7] and fatty acid binding protein 3 from Obs [8], the identities and functional roles of BMAd‐specific SASP components during physiological aging remain poorly defined. The bone marrow represents a unique tissue wherein skeletal and adipose components coexist within a shared anatomical compartment. This distinctive characteristic provides a distinct platform for investigating molecular interactions between BMAd‐derived SASP factors and other bone marrow cells. Elucidating these interactions may facilitate comprehensive management strategies for the complex microenvironment of aging bone marrow.

Chronic low‐grade inflammation represents a hallmark pathological feature of the aging bone marrow microenvironment. The cGAS‐STING signaling pathway, a mediator of DNA immune sensing, is widely recognized as a key driver of chronic inflammation and functional decline in aging [9, 10, 11, 12]. However, emerging evidence identifies STING as an immunoregulatory node that can modulate a unique, discrete tonic IFN‐I signal within osteoclast precursors [13]. Contrary to this pro‐inflammatory paradigm, STING‐deficient mice exhibit accelerated trabecular bone loss in the femur from birth through middle age (15‐month‐old) compared to wild‐type controls, whereas myeloid‐restricted STING signaling ameliorates postmenopausal bone loss by suppressing osteoclast formation [13]. Conversely, other studies have reported that pharmacological inhibition of STING signaling can improve postmenopausal bone loss [14] or enhance the osteogenic differentiation capacity of aged BMSCs [15]. Therefore, although STING signaling is extensively involved in bone development and remodeling activities, its precise role appears context‐dependent, leading to several critical unresolved questions: (1) Which specific cell populations within the multi‐lineage bone marrow microenvironment primarily express aging‐affected STING signaling, and what factors drive its expression? (2) How does the function of STING signaling in skeletal aging differ from its roles in bone development and maturation stages? (3) Is there a correlative or causative relationship between STING signaling and the formation of other characteristic pathological events in aged bone marrow, such as BMAT expansion?

In this study, we report significant activation of STING signaling within BMMs during natural aging in mice. BMM‐specific *Sting* knockout mice reversed the accelerated bone loss pattern observed around the young adult stage (3‐month‐old) when progressing from middle‐aged (10‐month‐old) to senescent (24‐month‐old) stages. Furthermore, this phenotypic rescue was consistent with the delayed bone loss observed in wild‐type mice following adeno‐associated virus 9 (AAV9)‐mediated specific knockdown of *Sting* in BMMs from middle‐aged to old (18‐month‐old) stages. Mechanistically, soluble DPP4 (sDPP4), secreted by senescent BMAds that accumulate during skeletal aging, functions as an extracellular ligand for Toll‐like receptor 4 (TLR4) on BMMs. It thereby cooperates with endogenous mitochondrial DNA (mtDNA) to activate STING signaling. Concurrently, the downstream CD38‐mediated nicotinamide adenine dinucleotide (NAD^+^) salvage synthesis pathway is activated. STING signaling activation directly upregulates CD38 expression, an event that subsequently promotes bone marrow inflammation. In addition, via its ecto‐NADase activity, heightened CD38 expression reduces local nicotinamide mononucleotide (NMN) availability, ultimately impairing the osteogenic differentiation capacity of BMSCs. Critically, pharmacological inhibition of STING combined with NMN supplementation effectively maintained skeletal health from middle age onward. In conclusion, these findings demonstrate that specific inhibition of BMM STING signaling represents a potential therapeutic strategy for delaying skeletal aging.

## 2 Results

### 2.1 Age‐Dependent Activation of STING Signaling in BMMs During Skeletal Aging

Initial assessment of STING signaling activation during skeletal aging revealed a gradual, age‐dependent increase in cytoplasmic double‐stranded DNA (dsDNA) and the second messenger cyclic guanosine monophosphate (cGAMP) levels in bone marrow of 3‐, 10‐, 18‐, and 24‐month‐old male mice (Figure 1a). Correspondingly, phosphorylation levels of STING and its canonical downstream targets (TBK1 and IRF3), along with non‐canonical NF‐κB phosphorylation, showed progressive elevation in aged bone marrow (Figure 1b, Figure S1a), indicating STING signaling activation as a characteristic feature of skeletal aging. Despite confirming pathway activation in aged bone marrow, the primary cellular contributors remained unclear. Therefore, comparative analysis of *Sting* mRNA levels across key bone marrow populations, including BMMs, pre‐osteoclasts (pre‐Ocs), Ocs, BMSCs, Obs, neutrophils (Neu), and BMAds, from young adult mice demonstrated that BMMs exhibited the highest basal *Sting* expression among all examined cell types (Figure 1c). Furthermore, *Sting* mRNA levels were upregulated in BMMs from 24‐month‐old mice (24 M‐BMMs) compared to those from 3‐month‐old mice (3 M‐BMMs) (Figure 1d). To extend these findings, a previously published single‐cell RNA sequencing (scRNA‐seq) dataset, encompassing bone marrow cells from 1‐, 1.5‐, 3‐, and 16‐month‐old mice [1], revealed that *STING* is broadly expressed in BMMs and demonstrates age‐dependent alterations compared to mesenchymal lineage cells in the bone marrow (Figure 1e,f). We next examined STING phosphorylation levels in BMMs as a function of age by comparing 3 M‐BMMs, BMMs from 10‐month‐old mice (10 M‐BMMs), BMMs from 18‐month‐old mice (18 M‐BMMs), and 24 M‐BMMs. As anticipated, progressive elevation in STING phosphorylation was observed in BMMs during aging (Figure 1g,h). Notably, the stable total STING protein level likely reflects a dynamic equilibrium between increased mRNA‑driven synthesis and autophagy‑mediated degradation [16]. Under this high‑turnover state, the age‑related accumulation of p‑STING is more plausibly attributable to enhanced upstream signaling rather than alterations in total protein abundance. In situ fluorescence staining using antibodies against p‐STING and F4/80 revealed a significant increase in p‐STING‐positive macrophages within the femoral bone marrow of senescent mice (Figure 1i, Figure S1b).

**FIGURE 1:**
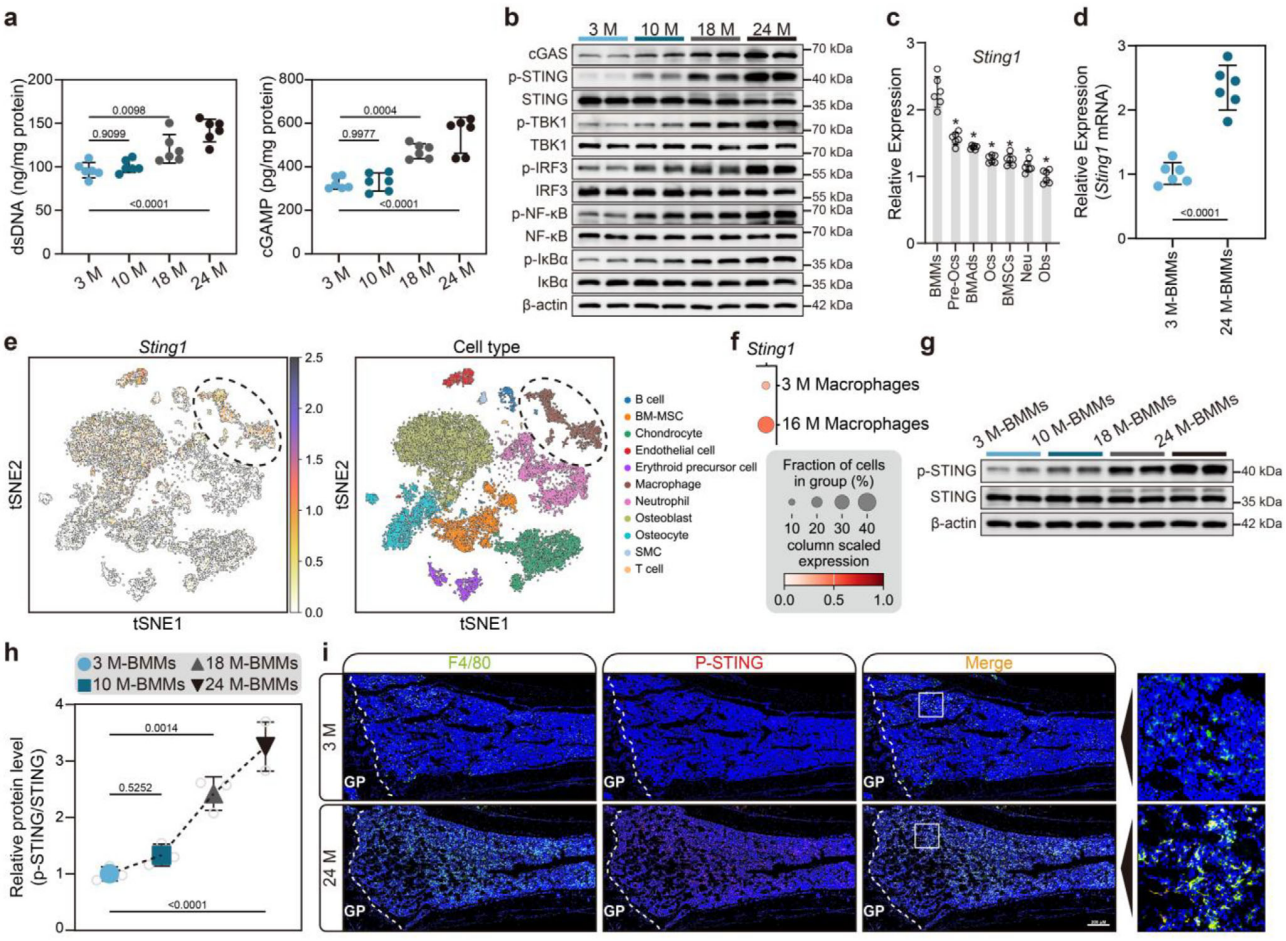
Progressive activation of STING signaling in BMMs during skeletal aging. (a) dsDNA and cGAMP concentrations in femoral bone marrow from 3‐, 10‐, 18‐, and 24‐month‐old male mice (*n* = 6 biological replicates). (b) Representative immunoblots of cGAS, STING, TBK1, IRF3, NF‐κB, and IκB protein/phosphorylation levels in bone marrow from 3‐, 10‐, 18‐, and 24‐month‐old male mice (*n* = 3 biological replicates). (c) qPCR analysis of *Sting* mRNA in BMMs, pre‐Ocs, BMAds, Ocs, BMSCs, Neu, and Obs of 3‐month‐old mice (*n* = 6 biological replicates; * *p* < 0.05 compared to BMMs). (d) qPCR analysis of *Sting* mRNA in 3 M‐/24 M‐BMMs (*n* = 6 biological replicates). (e) Bioinformatics analysis of scRNA‐seq of bone marrow cells from 1‐, 1.5‐, 3‐, and 16‐month‐old male mice. (f) Dot plot with hierarchical cluster analysis of *STING* gene expression in 3‐month‐old and 16‐month‐old male mice. (g,h) Representative immunoblots of STING protein levels in 3, 10, 18, and 24 M‐BMMs (g) and quantification analysis (h) (*n* = 3 biological replicates). (i) Representative immunofluorescence staining showing that p‐STING was markedly detected in BMMs (F4/80) in femora from 24‐month‐old male mice (scale bar: 200 µm). Data are shown as mean ± SD.

### 2.2 Targeted STING Inhibition FROM Middle Age Onward Effectively Delays Skeletal Aging

To examine the functional impact of BMM STING signaling activation during skeletal aging, myeloid‐specific *Sting* knockout mice (*Sting^ΔMφ^*) were generated by crossing *Sting*‐floxed mice (*Sting^flox/flox^*) with *Lyz2*‐Cre mice (Figure S2a). Body mass of both 10‐month‐old and 18‐month‐old *Sting^ΔMφ^* mice remained comparable to their *Sting^flox/flox^* littermates (WT), thereby eliminating potential confounding effects of body weight on bone phenotypes (Figure S2b). Subsequent bone phenotyping via µCT revealed unexpected alterations in bone mass. *Sting^ΔMφ^* mice exhibited inferior, though statistically non‐significant, bone mass parameters prior to reaching 10‐month‐old stage, characterized by lower bone mineral density (BMD), trabecular bone volume (BV/TV), trabecular bone number (Tb.N), and trabecular thickness (Tb.Th), along with increased trabecular separation (Tb.Sp) (Figure 2a,b, Figure S2c). Strikingly, this trend began to reverse starting at the 10‐month‐old stage. By the 24‐month‐old stage, *Sting^ΔMφ^* mice demonstrated improved BV/TV, Tb. N, and Tb.Th, as well as reduced Tb. Sp compared to age‐matched WT mice, though these differences also did not reach statistical significance (Figure 2a,b). However, a comparison of the mean differences in bone mass changes specifically between the 10‐ and 24‐month‐old stages revealed a significantly attenuated rate of bone loss in *Sting^ΔMφ^* mice relative to WT (Figure S2d). Moreover, analysis of bone‐fat conversion in 24‐month‐old *Sting^ΔMφ^* mice by hematoxylin‐eosin (H&E) staining and calcein double‐labeling demonstrated a trend toward reduced marrow adipocyte numbers (Figure 2c, Figure S2e) and a modest elevation in the mineral apposition rate (MAR) and mineralizing surface per bone surface (MS/BS) (Figure 2d, Figure S2f) compared to WT. These results suggest that activation of STING signaling in BMMs plays a significant role in driving bone loss from middle age onward.

**FIGURE 2:**
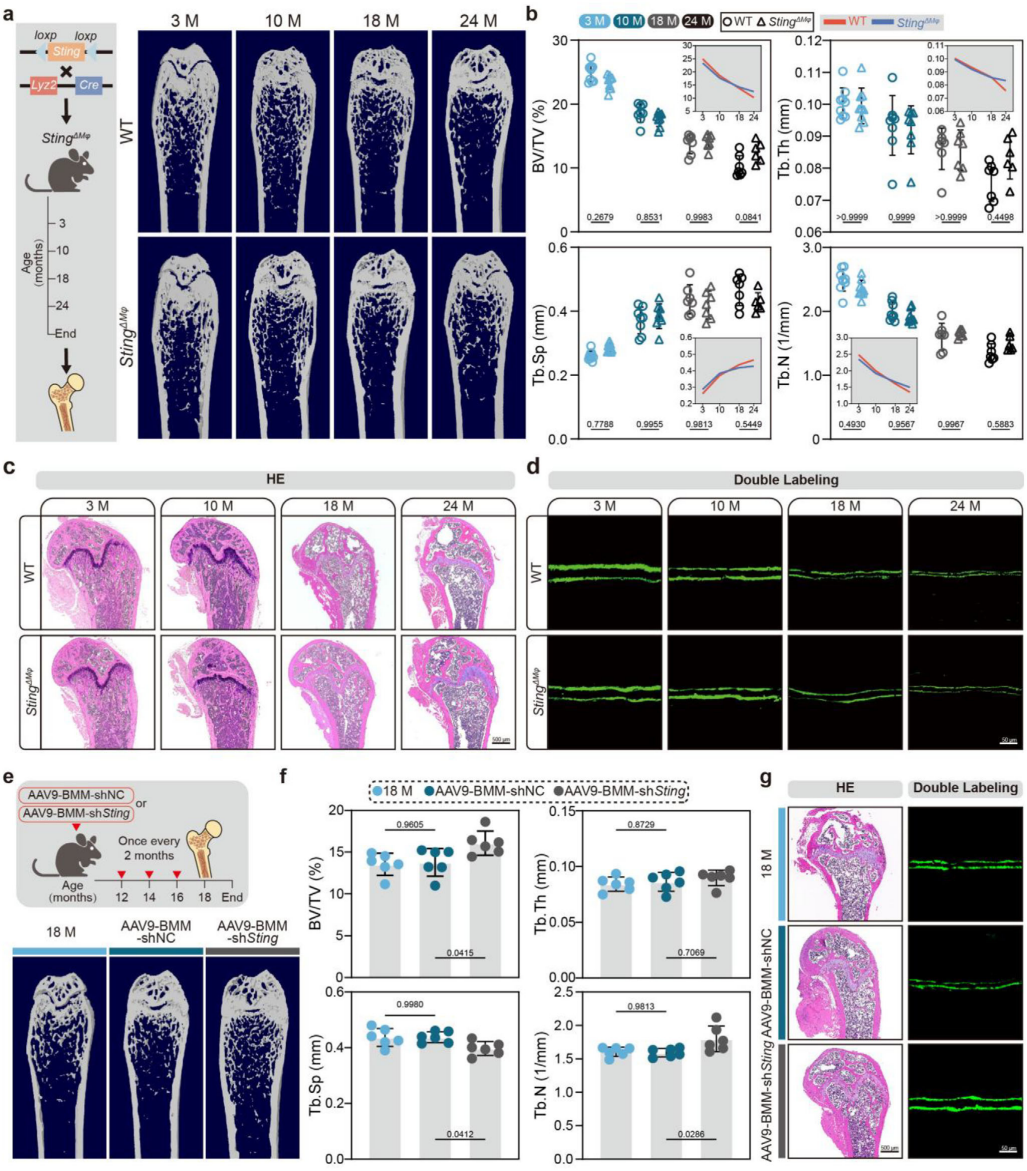
Targeted inhibition of BMM STING signaling during middle and old age effectively delays skeletal aging. (a, b) Representative micro‐CT images (a) and quantitative analysis of BV/TV, Tb. N, Tb.Th, and Tb.Sp (b) from 3‐, 10‐, 18‐, and 24‐month‐old male WT and *Sting^ΔMφ^* mice (*n* = 9 biological replicates for 3‐month‐old WT mice/*Sting^ΔMφ^* mice; *n* = 7 biological replicates for 10‐month‐old WT mice/*Sting^ΔMφ^* mice; *n* = 8 biological replicates for 18‐month‐old WT mice/*Sting^ΔMφ^* mice; *n* = 7 biological replicates for 24‐month‐old WT mice/*Sting^ΔMφ^* mice). (c) Representative images of H&E staining (scale bar: 100 µm). (d) Representative images of calcein double‐labeling (scale bar: 50 µm). (e,f) Representative micro‐CT images (e) and quantitative analysis of BV/TV, Tb. N, Tb.Th, and Tb.Sp (f) from AAV9‐BMM‐shNC‐treated or AAV9‐BMM‐sh*Sting*‐treated 18‐month‐old mice (*n* = 6 biological replicates). (g) Representative images of H&E staining (scale bar: 100 µm) and calcein double‐labeling (scale bar: 50 µm). Data are shown as mean ± SD.

A recent study reported that male *Sting^−/−^* mice exhibited a trend toward reduced bone mass at 6‐month‐old and 13‐month‐old, with statistically significant bone loss at 15‐month‐old compared to WT mice, while this phenomenon was not observed in female *Sting^−/−^* mice [13]. Consistent with these findings, we observed similar bone mass reduction in *Sting^ΔMφ^* mice before reaching 10 months old, but also identified a slower rate of bone loss after 10‐month‐old compared to WT mice. Importantly, to directly evaluate the therapeutic potential of the specific inhibition of *Sting* initiated in middle age, an AAV9 vector carrying short hairpin RNA (shRNA) targeting *Sting* under the *F4/80* promoter (AAV9‐BMM‐sh*Sting*) was employed for BMM‐specific knockdown (Figure 2e, Figure S2g). Consequently, AAV9‐BMM‐sh*Sting*‐treated 18‐month‐old mice displayed significantly delayed bone loss, evidenced by higher BMD, BV/TV, and Tb. N, coupled with reduced Tb.Sp, relative to age‐matched controls (Figure 2f, Figure S2h). Additionally, these mice with BMM‐specific *Sting* knockdown exhibited a markedly enhanced MAR and MS/BS, and a significant reduction in marrow adipocyte numbers (Figure 2g, Figure S2i,j).

### 2.3 Accumulation of BMAds Mediates BMM STING Signaling During Skeletal Aging

Since sexual dimorphism, as indicated in prior studies, could influence bone phenotypes in *Sting^−/−^* mice, it was essential to systematically evaluate the comprehensive impact of *Sting*‐specific deficiency on bone phenotypes in the late stages of skeletal aging under more integrated conditions. Therefore, a series of co‐culture models were established to simulate potential interactions within the complex bone marrow microenvironment during natural aging. Initially, to verify whether activation of STING signaling in BMMs from middle age onward in mice directly accelerates bone loss, BMMs from 18‐month‐old WT or *Sting^ΔMφ^* mice (WT‐ or *Sting^ΔMφ^*‐18 M‐BMMs) were co‐cultured with BMSCs from 3‐month‐old mice (3 M‐BMSCs), followed by assessment of osteogenic differentiation. When 3 M‐BMSCs were co‐cultured with WT‐18 M‐BMMs but not *Sting^ΔMφ^*‐18 M‐BMMs, a significant impairment was observed in alkaline phosphatase (ALP) activity and mineralization function of BMSCs (Figure S3a). In addition, the coupling of angiogenesis and osteogenesis plays a critical role in mammalian bone homeostasis [17, 18]. Similarly, compared to *Sting^ΔMφ^*‐18 M‐BMMs, the tube formation ability in bEnd.3 cells was found to be impaired when co‐cultured with WT‐18 M‐BMMs (Figure S3b). Consequently, the combined effects of these factors may mask the negative impacts of STING signaling deficiency on bone homeostasis during development and early aging stages. The chronic pathological transformation of the bone marrow microenvironment during skeletal aging might exert reciprocal effects on the activation of BMM STING signaling; thus, a systematic investigation of potential exogenous mediating factors will be essential to delineate this bidirectional regulatory axis.

Chronic inflammation, a hallmark of the aging bone marrow microenvironment from middle age onward, was initially examined as a potential inducer of STING signaling in BMMs. Young adult male mice were treated with a sublethal dose of LPS or PBS for four consecutive weeks. LPS‐treated mice showed elevated levels of dsDNA and cGAMP (Figure 3a) along with activated STING signaling in BMMs (Figure 3b, Figure S3c). Additionally, higher endotoxin levels were detected in both serum and bone marrow supernatants from 24‐month‐old mice compared to 3‐month‐old mice (Figure 3c). To further demonstrate the association between BMM STING signaling activation and bone marrow inflammation, 3 M‐BMMs were cultured with bone marrow supernatants from either 3‐month‐old (3 M‐SN) or 18‐month‐old (18 M‐SN) mice (Figure 3d). Treatment with 18 M‐SN, relative to 3 M‐SN, increased both dsDNA and cGAMP levels (Figure 3d) and promoted STING signaling activation in 3 M‐BMMs (Figure 3e, Figure S3d).

**FIGURE 3:**
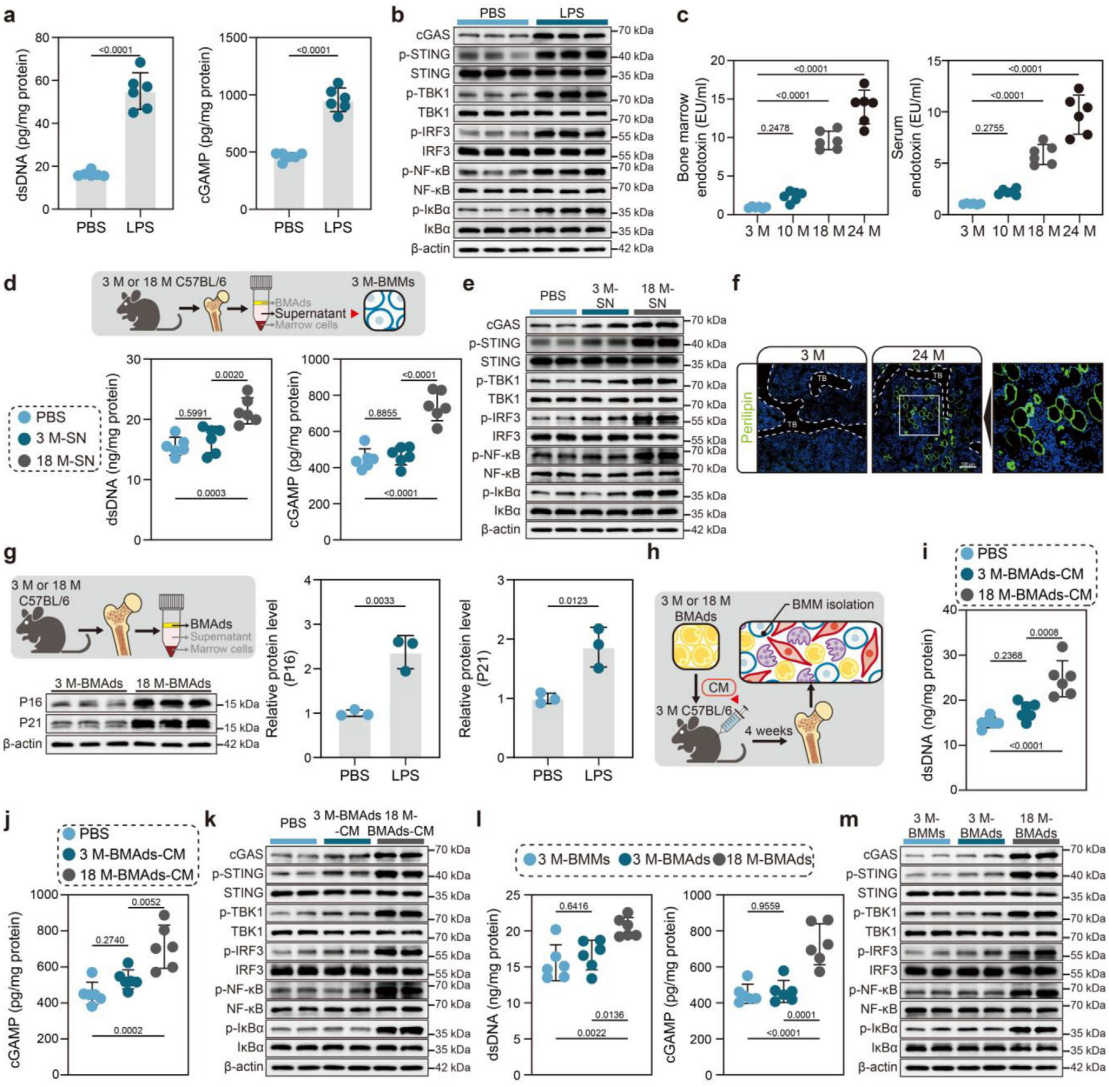
Accumulation of BMAds during skeletal aging activates BMM STING signaling. (a, b) Intracellular dsDNA and cGAMP concentrations (*n* = 6 biological replicates) (a) and representative immunoblots of cGAS, STING, TBK1, IRF3, NF‐κB, and IκB protein/phosphorylation levels (*n* = 3 biological replicates) (b) in BMMs from PBS‐treated or LPS‐treated male mice. (c) Endotoxin levels in serum and femoral bone marrow from 3‐, 10‐, 18‐, and 24‐month‐old male mice (*n* = 6 biological replicates). (d,e) dsDNA and cGAMP concentrations (*n* = 6 biological replicates) and representative immunoblots of cGAS, STING, TBK1, IRF3, NF‐κB, and IκB protein/phosphorylation levels (*n* = 3 biological replicates) (e) in BMMs from PBS‐treated or 3 M‐/18 M‐SN‐treated male mice. (f) Representative immunofluorescence staining and H&E staining of BMAds in femoral sections from 24‐month‐old male mice (scale bar: 100 µm). (g) Representative immunoblots of P16 and P21 protein levels in 3 M‐/18 M‐BMAds (*n* = 3 biological replicates). (h) Schematic of the treatment of 3‐month‐old male mice with 3 M‐/18 M‐BMAds‐CM related to data shown in (i–k). i–k dsDNA concentration (*n* = 6 biological replicates) (i), cGAMP concentration (*n* = 6 biological replicates) (j), and representative immunoblots of cGAS, STING, TBK1, IRF3, NF‐κB, and IκB protein/phosphorylation levels (*n* = 3 biological replicates) (k). (l,m) dsDNA and cGAMP concentrations (*n* = 6 biological replicates) (l) and representative immunoblots of cGAS, STING, TBK1, IRF3, NF‐κB, and IκB protein/phosphorylation levels (*n* = 3 biological replicates) (m) in BMMs from a co‐culture system. Data are shown as mean ± SD.

In further support of this, another characteristic feature of aged bone is the progressive expansion of BMAT that accompanies bone loss. Recent evidence indicates that glucocorticoid‐induced senescence of BMAds can propagate secondary senescence through the acquisition of a SASP [5]. Consistent with this mechanism, natural accumulation of perilipin‐positive BMAds was observed in senescent mice (Figure 3f). To determine whether this process associates with BMM STING signaling, BMAds were isolated from 18‐month‐old (18 M‐BMAds) and 3‐month‐old (3 M‐BMAds) mice using an established centrifugation‐based method (Figure 3g). Strikingly, 18 M‐BMAds demonstrated elevated protein levels of P16 and P21 compared to 3 M‐BMAds, indicating accumulation of senescent properties (Figure 3g). To investigate the possibility that BMM STING signaling activation is partially driven by SASP factors, conditioned medium from either 3 M‐BMAds (3 M‐BMAds‐CM) or 18 M‐BMAds (18 M‐BMAds‐CM) was injected into the bone marrow cavity of 3‐month‐old mice, and BMM STING signaling activation was measured (Figure 3h). Compared to 3 M‐BMAds‐CM, injection of 18 M‐BMAds‐CM increased dsDNA and cGAMP levels (Figure 3i,j) and enhanced BMM STING signaling activation (Figure 3k, Figure S3e). Subsequently, an in vitro co‐culture system was established, in which 3 M‐BMMs were incubated with either 3‐ or 18 M‐BMAds. Corroborating the in vivo findings, co‐culture with 18 M‐BMAds resulted in increased dsDNA and cGAMP levels (Figure 3l) along with activated STING signaling (Figure 3 m, Figure S3f) in BMMs. Thus, these data collectively indicate that the natural BMAds accumulation during aging likewise promotes BMM STING signaling activation. Given the significant role of BMAds accumulation in BMM STING signaling activation, potential SASP factors derived from BMAds were further explored.

### 2.4 Senescent BMAd‐Derived sDPP4 to Activate BMM STING Signaling

To identify key SASP factors mediating BMAd‐driven skeletal aging, 4D‐label‐free quantitative proteomic analysis was performed to detect differentially expressed factors in 3 M‐SN and 18 M‐SN from rats. This screening revealed 44 differentially expressed secretory proteins exhibiting ≥ 1.5‐fold upregulation in 18 M‐SN (Figure 4a). Subsequently, cross‐analysis with a published BMAd‐specific RNA‐seq dataset (ENA: ERP013883) [19] identified DPP4 as the sole significantly altered factor common to both datasets (Figure 4b). Previous studies have established DPP4 as a novel pro‐inflammatory adipokine linking adipose tissue expansion to increased inflammation, insulin resistance, and metabolic syndrome [19, 20]. Whereas conditioned media from 3‐month‐old neutrophils (Neu‐CM), BMMs (BMMs‐CM), pre‐Ocs (pre‐Ocs‐CM), Ocs (Ocs‐CM), BMSCs (BMSCs‐CM), and Obs (Obs‐CM) contained minimal sDPP4, substantially higher sDPP4 levels were detected in BMAds‐conditioned media (BMAds‐CM) (Figure 4c). This BMAd‐specific expression was further confirmed by perilipin and DPP4 immunofluorescence co‐localization (Figure 4d). Critically, compared to 3 M‐BMAds‐CM, sDPP4 levels were significantly higher in conditioned media from aged BMAds (18 M‐BMAds‐CM) relative to young controls (3 M‐BMAds‐CM) (Figure 4e). These findings suggest sDPP4 may contribute to chronic alterations in the bone marrow microenvironment during aging.

**FIGURE 4:**
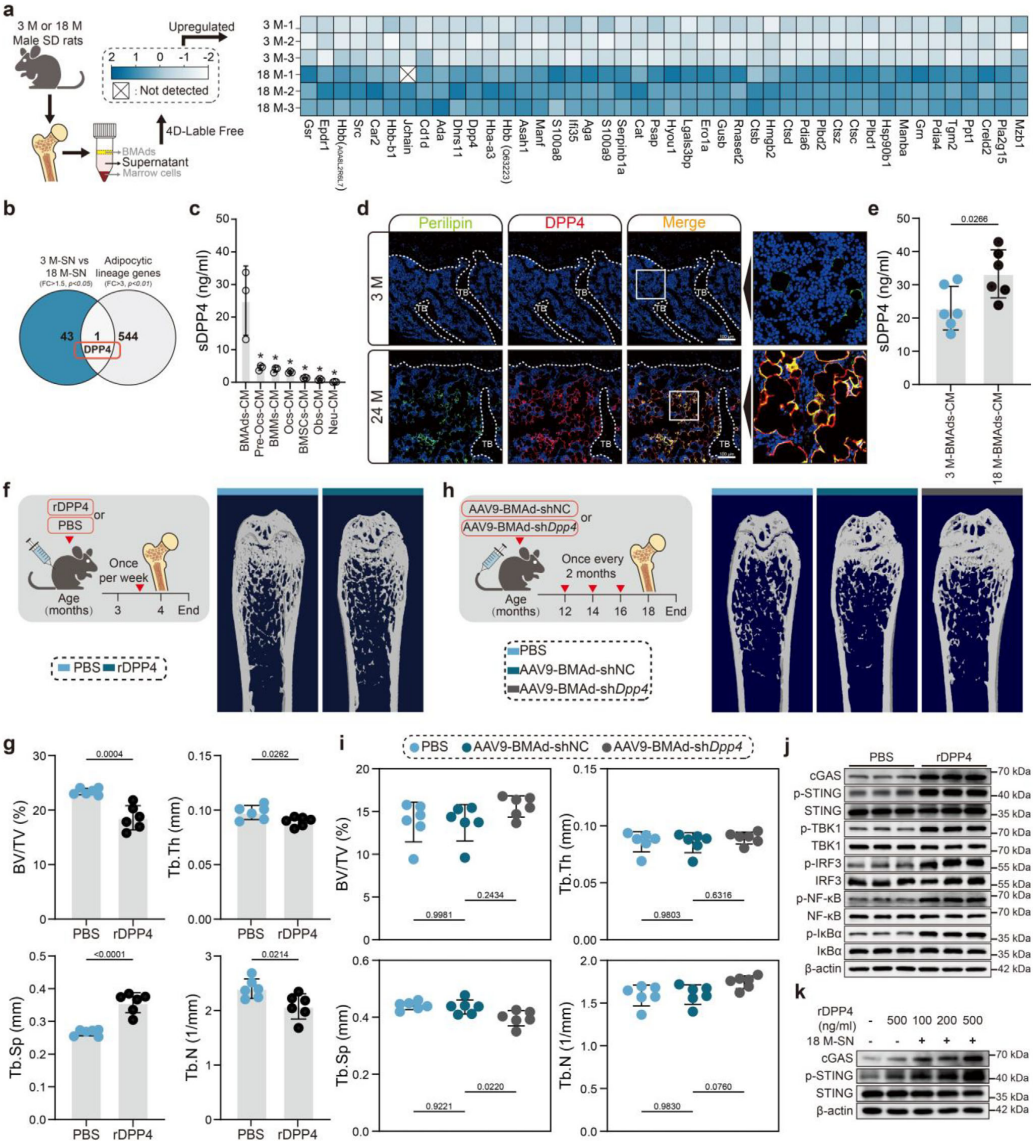
BMAd accumulation and sDPP4 release during skeletal aging activate BMM STING signaling. (a) Heatmap of dysregulated factors (≥1.5‐fold change) in the bone marrow supernatant of 3‐/18‐month‐old male rats identified through 4D label‐free technology (*n* = 3 biological replicates). (b) Venn diagram of dysregulated factors in the bone marrow of rats. (c) ELISA of sDPP4 levels in conditioned medium collected from BMAds, Pre‐Ocs, BMMs, Ocs, BMSCs, Obs, and Neu (*n* = 6 biological replicates; *p < 0.05 compared to BMAds‐CM). (d) Representative immunofluorescence staining showing that DPP4 was markedly detected in BMAds (Perilipin) in femora from 3‐/24‐month‐old male mice (scale bar: 100 µm). (e) ELISA of sDPP4 levels in conditioned medium collected in 3 M‐/18 M‐BMAds (*n* = 6 biological replicates). (f,g) Representative micro‐CT images (f) and quantitative analysis of BV/TV, Tb. N, Tb.Th, and Tb.Sp (g) from PBS‐treated or rDPP4‐treated male mice (*n* = 6 biological replicates). (h,i) Representative micro‐CT images (h) and quantitative analysis of BV/TV, Tb. N, Tb.Th, and Tb.Sp (i) from AAV9‐BMAd‐shNC‐treated or AAV9‐BMAd‐sh*Dpp4*‐treated male mice (*n* = 6 biological replicates). (j) Representative immunoblots of cGAS, STING, TBK1, IRF3, NF‐κB, and IκB protein/phosphorylation levels in BMMs from PBS‐treated or rDPP4‐treated male mice (*n* = 3 biological replicates). (k) Representative immunoblots of cGAS and STING protein/phosphorylation levels in BMMs treated with either rDPP4 alone or in combination with 18 M‐SN (*n* = 3 biological replicates). Data are shown as mean ± SD.

To evaluate the impact of DPP4 on skeletal aging, human recombinant DPP4 (rDPP4) or PBS was administered weekly via intramedullary injection into 3‐month‐old mice for four weeks before analyzing their bone phenotypes (Figure 4f). µCT analysis revealed that rDPP4‐treated mice exhibited lower BMD, BV/TV, Tb. N, and Tb.Th, along with higher Tb.Sp compared to PBS‐treated controls, suggesting elevated DPP4 levels may disrupt skeletal homeostasis (Figure 4g, Figure S4a). Conversely, AAV9 bearing the *Pdgfrα* promoter (AAV9‐BMAd‐sh*Dpp4*) was employed to target *Dpp4* expression in BMAds (Figure S4b). This intervention resulted in significantly reduced Tb. Sp and non‐significant increases in BMD, BV/TV, Tb. N, and Tb.Th (Figure 4h,i, Figure S4c). Furthermore, the effect of DPP4 elevation on BMM STING signaling was examined. After isolating BMMs, we observed significantly activated STING signaling in BMMs from rDPP4‐injected mice compared to PBS‐injected controls (Figure 4j, Figure S4d). Notably, four weeks of rDPP4 treatment significantly increased endotoxin levels in the bone marrow of young mice compared with the PBS control group (Figure S4e), suggesting that chronic rDPP4 exposure induces local inflammatory alterations in the bone marrow microenvironment. Consistent with these findings, while rDPP4 treatment alone did not significantly activate STING signaling in BMMs in vitro, its combination with 18 M‐SN (SN‐rDPP4), containing higher endotoxin levels, enhanced STING signaling activation in an rDPP4 dose‐dependent manner (Figure 4k, Figure S4f).

### 2.5 sDPP4 Binds TLR4 and Cooperates with mtDNA for STING Signaling Co‐Activation

To identify the functional receptor for sDPP4 in BMMs, three previously reported DPP4‐binding receptors, protease‐activated receptor 2 (PAR2) [21], insulin‐like growth factor 2 receptor (IGF2R) [22], and caveolin‐1 [23], were systematically evaluated. Notably, neither PAR2 inhibition with GB83, competition of DPP4‐IGF2R interaction with excess free mannose‐6‐phosphate, nor caveolin‐1 knockdown using adenoviruses (Ad)‐mediated shRNA significantly altered SN‐rDPP4‐induced STING phosphorylation (Figure S5a). STING activation requires endoplasmic reticulum‐to‐endoplasmic reticulum‐Golgi intermediate compartment translocation mediated by transforming growth factor beta‐activated kinase 1 (TAK1)‐binding protein 1 (TAB1), a process potentiated by TLR agonist‐induced TAK1 activation [24]. Considering the observed synergistic, dose‐dependent STING activation by rDPP4 combined with high‐endotoxin 18 M‐SN (Figure 5a), TLRs were hypothesized as candidate receptors. Consistently, rDPP4 treatment increased TLR2/4 expression in BMMs to varying degrees, while SN‐rDPP4 treatment induced more substantial TLR2/4 upregulation (Figure 5b, Figure S5b). Alongside this, enhanced TAK1 and STING phosphorylation were observed following SN‐rDPP4 treatment, which was partially attenuated by either TLR2 inhibitor C29 or TLR4 inhibitor resatorvid (Figure 5c, Figure S5c). We further treated 18‐month‐old mice with resatorvid for two weeks and assessed STING activation in isolated BMMs. Compared with PBS administration, resatorvid significantly reduced p‐STING levels in BMMs from 18‐month‐old mice (Figure S5d).

**FIGURE 5:**
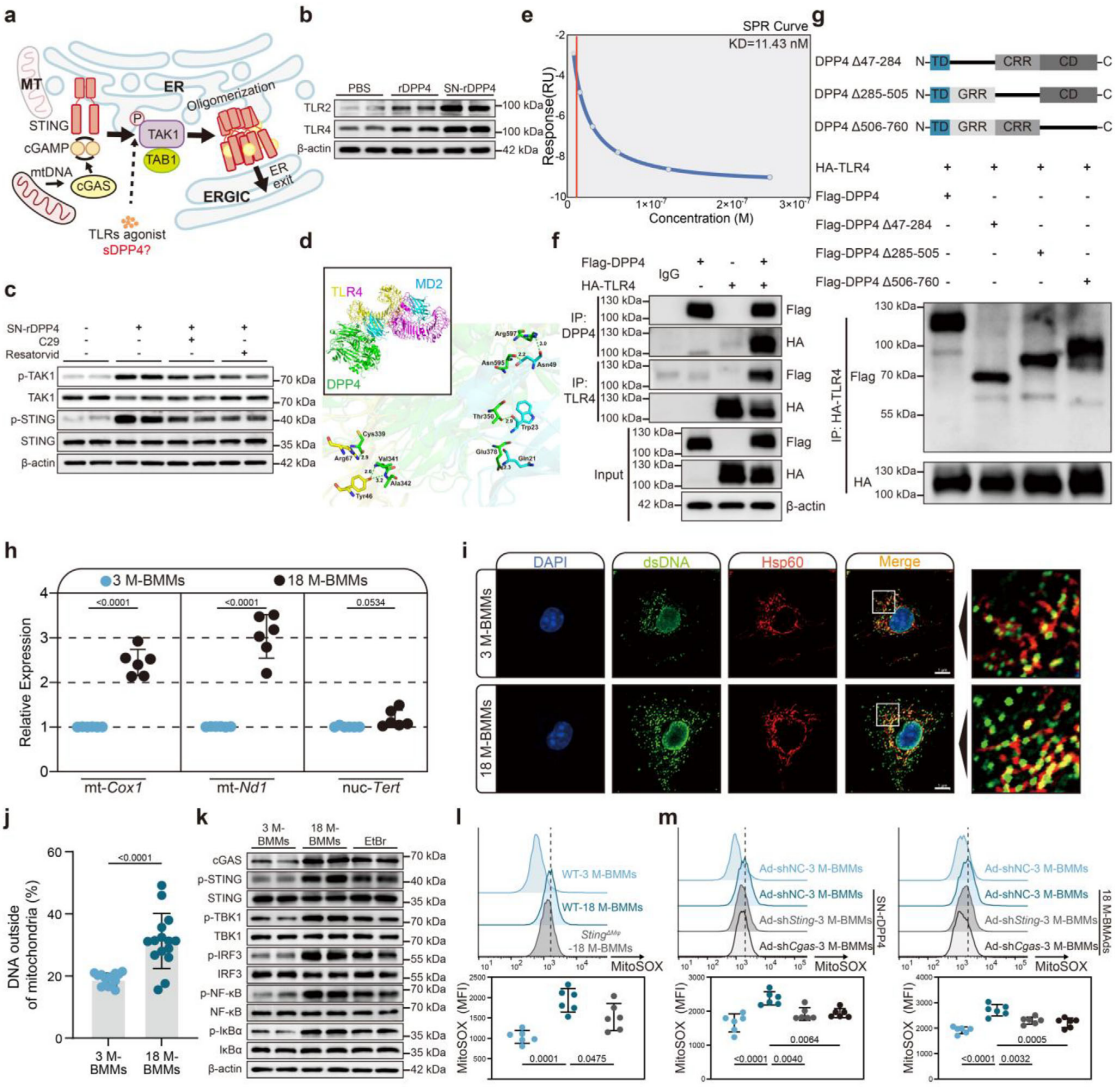
BMAd‐derived sDPP4 activates BMM STING signaling through TLR4 binding and mtDNA cooperation. (a) Schematic diagram of sDPP4‐mediated STING activation. (b) Representative immunoblots of TLR2 and TLR4 protein levels in BMMs from rDPP4‐treated or SN‐rDPP4‐treated male mice (*n* = 3 biological replicates). (c) Representative immunoblots of TAK1 and STING protein/phosphorylation levels in BMMs stimulated with SN‐rDPP4 and treated with C29 or Resatorvid (*n* = 3 biological replicates). (d) Molecular docking analysis of the direct binding mode between the TLR4/MD‐2 complex and the DPP4 structure. (e) SPR analysis of the direct interaction between rDPP4 and rTLR4. (f) Co‐Immunoprecipitation analysis of Flag‐*Dpp4* and HA‐*Tlr4* binding. (g) Co‐Immunoprecipitation analysis of DPP4 deletion mutants binding to full‐length TLR4. (h) qPCR analysis of cytosolic DNA extracted from the cytosolic extracts of 3 M‐/24 M‐BMMs (*n* = 6 biological replicates). (i,j) Representative immunofluorescence staining of s anti‐dsDNA (green), anti‐Hsp60 (red), and DAPI (blue) in 3 M‐/18 M BMMs (scale bar, 1 µm) (i). The percentage of dsDNA (green) outside of mitochondria (red) was quantified (j) (*n* = 15 from 3 biological replicates). (k) Representative immunoblots of cGAS, STING, TBK1, IRF3, NF‐κB, and IκB protein/phosphorylation levels in BMMs treated with EtBr (*n* = 3 biological replicates). (l) Flow cytometry analysis and quantification of mtROS production in WT‐3 M‐/WT‐18 M‐BMMs and *Sting^ΔMφ^*‐18 M‐BMMs (*n* = 6 biological replicates). (m) Flow cytometry analysis and quantification of mtROS production in SN‐rDPP4‐treated or co‐cultured BMMs with or without *Cgas* and *Sting* knockdown (*n* = 6 biological replicates). Data are shown as mean ± SD.

Given the observed more significant reductions in TAK1 and STING phosphorylation following resatorvid treatment, TLR4 was selected for subsequent investigation. Initial protein docking analysis was performed to investigate the interaction between rDPP4 and TLR4, using the TLR4/myeloid differentiation protein 2 (MD2) complex structure (PDB ID: 3FXI) alongside the DPP4 structure (PDB ID: 1J2E) (Figure 5d). Surface plasmon resonance (SPR) assays were conducted to further evaluate the interaction between rDPP4 and the recombinant extracellular domain of TLR4 (rTLR4). The results demonstrated concentration‐dependent resonance responses, showing specific binding of rDPP4 (7.8125–250 nm) to rTLR4 with an equilibrium dissociation constant (KD) of 11.43 nm (Figure 5e). While these SPR sensorgrams displayed characteristic negative signals, this is a phenomenon potentially associated with aptamer conformational transitions [25]. Furthermore, co‐immunoprecipitation experiments in 293T cells transfected with FLAG‐*Dpp4* and HA‐*Tlr4* plasmids further confirmed the intracellular interaction between DPP4 and TLR4 (Figure 5f).

To further identify the precise binding domain of DPP4 with TLR4, DPP4 mutants were systematically generated with deletions in specific regions: the glycosylation‐rich domain (nucleotides 47–284), cysteine‐rich domain (nucleotides 285–505), and catalytic domain (nucleotides 506–760). Notably, none of these single‐domain deletions substantially affected its binding to TLR4 (Figure 5g), suggesting that multiple DPP4 domains may simultaneously engage with TLR4 and that single‐domain deletions did not markedly impact their interaction. Collectively, these results demonstrate that TLR4 directly functions as a functional receptor for BMAd‐derived sDPP4, thereby facilitating STING signaling activation.

Beyond the extrinsic mechanism mediated by BMAd‐derived sDPP4, the potential contribution of intrinsic factors to STING signaling activation was investigated. Since the cGAS‐STING signaling functionally detects the presence of cytosolic DNA [26], whether skeletal aging leads to increased cytosolic DNA in BMMs was systematically examined. Cytosolic DNA may theoretically originate from either nuclear or mitochondrial sources. DNA purification from cytoplasmic extracts revealed that cytosolic DNA in 18 M‐BMMs primarily consisted of mtDNA, as evidenced by elevated levels of *Nd1* and *Cox1*, rather than nuclear DNA (nDNA) indicated by *Tert* (Figure 5h). Consistent with this, 18 M‐BMMs showed significantly increased cytosolic dsDNA outside mitochondria compared to 3 M‐BMMs, supporting aging‐induced mtDNA release in BMMs (Figure 5i,j). To further investigate whether mtDNA release into the cytoplasm causes BMM STING signaling activation, an established protocol was applied using low‐concentration ethidium bromide (EtBr) to deplete cellular mtDNA [27]. As expected, mtDNA depletion substantially reduced the increases in cGAS and p‐STING levels in BMMs (Figure 5k, Figure S5e). Due to the absence of repair proteins and protective histones, mtDNA is more vulnerable to mitochondrial reactive oxygen species (mtROS), byproducts of mitochondrial respiration [28]. Additionally, increased mtROS production was observed in BMMs from 18‐month‐old WT (WT‐18 M) mice (WT‐18 M‐BMMs) compared to WT‐3 M‐BMMs, whereas BMMs from 18‐month‐old *Sting^ΔMφ^* (*Sting^ΔMφ^*‐18 M) mice (*Sting^ΔMφ^*‐18 M‐BMMs) exhibited decreased mtROS levels (Figure 5l). Similarly, in vitro treatment with SN‐rDPP4 or co‐culture with 18 M‐BMAds enhanced mtROS generation in 3 M‐BMMs, an effect prevented by *Cgas* or *Sting* knockdown in BMMs (Figure 5m, Figure S5f).

### 2.6 STING Signaling Activation in Aging BMMs Correlates With NAD^+^ Homeostasis Dysregulation

Subsequently, the downstream consequences of STING signaling triggered by excessive mtDNA accumulation were investigated in BMMs. Previous reports indicate that inflammatory activation in macrophages involves mtROS‐induced DNA damage and NAD^+^ depletion [29]. NAD^+^ serves as an essential coenzyme in redox reactions and acts as a substrate in non‐redox enzymatic processes including cellular signaling, DNA repair, and epigenetic modifications, thereby regulating health‐span and aging [30]. A progressive decline in NAD^+^ levels was observed with advancing age (Figure 6a). Since NAD^+^ metabolism primarily involves three main pathways: the Preiss‐Handler pathway, *de novo* synthesis pathway, and salvage pathway (Figure 6b), and considering the absence of nicotinic acid (NA) but the presence of nicotinamide (NAM) in the culture medium, NAD^+^ could be derived from tryptophan via the *de novo* pathway or recycled from NAM via the salvage pathway. Therefore, the mRNA expression of key enzymes involved in both the *de novo* synthesis pathway (*Ido1*, *Afmid*, *Kyat1*, *Kynu*, *Kmo*, *Haao*, *Qprt*) and the salvage pathway (*Cd38*, *Nampt*, *Nmnat1 ‐ 3*, *Parp1 ‐ 2*, *Sirts*) in BMMs from different‐aged mice was quantified. While gene expression encoding key enzymes for NAD^+^ synthesis through the *de novo* pathway showed minimal changes (Figure S6a,b), significant increases were detected in mRNA levels of salvage pathway enzymes *Cd38*, *Nampt*, *Nmnat1*, and *Nmnat3*, accompanied by decreased *Sirt1*, *Sirt3*, and *Sirt5* mRNA levels (Figure 6b,c). These transcriptional changes were reflected at the protein level (Figure 6d, Figure S6c), indicating that the salvage synthesis pathway that compensates for NAD^+^ fluctuations becomes progressively activated in BMMs during skeletal aging and co‐occurs with BMM STING signaling activation.

**FIGURE 6:**
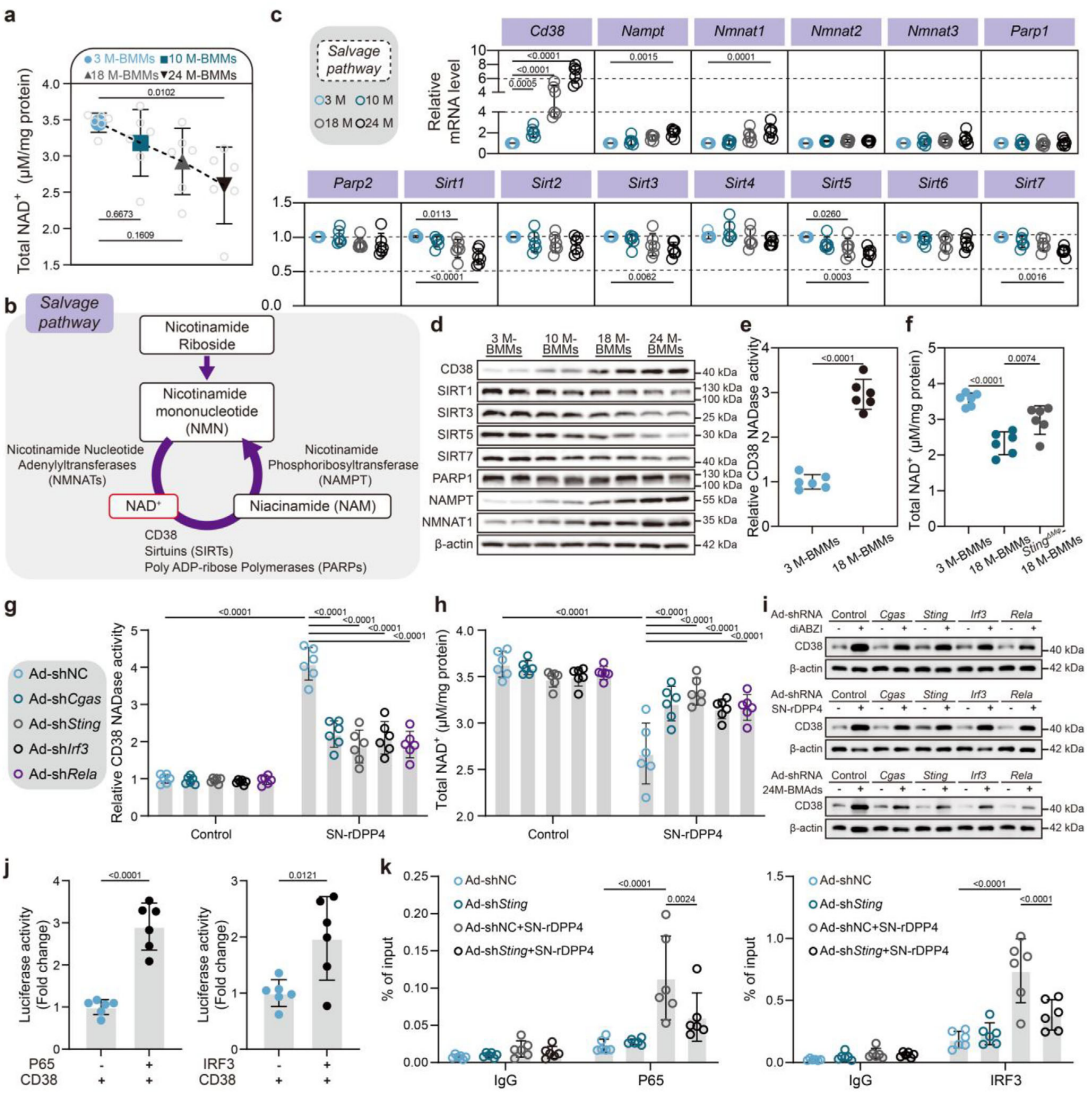
STING signaling promotes NAD^+^ decline in BMMs during skeletal aging by enhancing *Cd38* transcription through IRF3 and P65. (a) WST‐8 analysis of NAD levels in 3, 10, 18, and 24 M‐BMMs (*n* = 6 biological replicates). (b) Schematic of key enzymes in the NAD salvage biosynthesis pathway. (c,d) qPCR (*n* = 6 biological replicates) and Western blotting (*n* = 3 biological replicates) analyses of key enzymes in the NAD^+^ salvage biosynthesis pathway in 3, 10, 18, and 24 M‐BMMs. (e) Fluorometric assay for CD38 NADase activity in 3 M‐/18 M‐BMMs (*n* = 6 biological replicates). (f) WST‐8 analysis of NAD^+^ levels in WT‐3 M‐/WT‐18 M‐BMMs and *Sting^ΔMφ^*‐18 M‐BMMs (*n* = 6 biological replicates). (g,h) Fluorometric assay and WST‐8 analysis of CD38 NADase activity (g) or NAD^+^ levels (h) in SN‐rDPP4‐treated BMMs with or without *Cgas*, *Sting*, Irf3, and *Rela* knockdown (*n* = 6 biological replicates). (i) Representative immunoblots of CD38 protein levels in diABZI‐treated, SN‐rDPP4‐treated, or co‐cultured BMMs with or without *Cgas*, *Sting*, Irf3, and *Rela* knockdown (*n* = 3 biological replicates). (j) Dual luciferase reporter assay in HEK 293T cells co‐transfected with CD38 luciferase reporter, IRF3 or P65 plasmid separately, and pRL‐CMV‐Renilla (*n* = 6 biological replicates). (k) Chromatin immunoprecipitation assay showing that IRF3 or P65 bound to the CD38 promoter in BMMs. Binding levels were increased in BMMs treated with SN‐rDPP4 and were abolished by *STING* knockdown (*n* = 6 biological replicates). Data are shown as mean ± SD.

Notwithstanding the demonstrated co‐activation of the STING signaling and the NAD^+^ salvage synthesis pathway in BMMs, their mechanistic relationship remains unclear. Since the expression of CD38 was significantly upregulated in senescent bone and the CD38 inhibitor 1 (78c) effectively prevented NAD^+^ decline in 18 M‐BMMs (Figure S6d), this suggests that 18 M‐BMMs may possess higher CD38 NADase activity than 3 M‐BMMs. To test this hypothesis, ε‐NAD, a modified NAD^+^ analog yielding time‐dependent fluorescence at 410 nm upon hydrolysis, was employed. Results demonstrated significantly enhanced CD38 NADase activity in lysates from 18 M‐BMMs compared to 3 M‐BMMs (Figure 6e). Critically, partial restoration of total NAD^+^ levels occurred in *Sting^ΔMφ^*‐18 M‐BMMs (Figure 6f), indicating a potential association between STING activation and CD38 NADase activity. To verify this, 3 M‐BMMs were treated with several STING‐activating conditions, including the STING agonist diABZI, SN‐rDPP4, or co‐culture with 18 M‐BMAds. These interventions universally induced varying degrees of NAD^+^ reduction and enhanced CD38 NADase activity in 3 M‐BMMs (Figure 6g,h, Figure S6e–g), accompanied by increased CD38 protein (Figure 6i, Figure S6h) and *Cd38* mRNA (Figure S6i) expression. Notably, all alterations were reversed by *cgas* or *Sting* knockdown (Figure 6g–i, Figure S6e–i), establishing that STING signaling directly upregulates CD38‐mediated NAD^+^ catabolism during skeletal aging.

The function of STING signaling primarily depends on its downstream transcription factors IRF3 and P65. Critically, *Irf3* or *Rela* knockdown (Figure S6j) partially restored the decreased NAD^+^ levels and attenuated the enhanced CD38 NADase activity in 3 M‐BMMs treated with either the STING agonist diABZI treatment, SN‐rDPP4 treatment, or co‐culture with 18 M‐BMAds (Figure 6g,h, Figure S6g). Concurrently, these interventions also suppressed the increased CD38 protein (Figure 6i, Figure S6h) and *Cd38* mRNA expression (Figure S6i), indicating that both IRF3 and P65 collectively mediate STING‐dependent regulation of CD38. Mechanistically, bioinformatic analysis of the *Cd38* 5' untranslated region revealed multiple putative binding sites for IRF3 and P65 (Figure S6k). This prediction was functionally validated by dual‐luciferase reporter assays in HEK 293T cells, where both transcription factors significantly enhanced Cd38 promoter activity (Figure 6j). In addition, chromatin immunoprecipitation (ChIP) analysis confirmed direct binding of IRF3 and P65 to the *Cd38* promoter region in BMMs (Figure 6k). This binding was triggered by diABZI treatment, SN‐rDPP4 treatment, or co‐culture with 18 M‐BMAds, and was suppressed by *STING* knockdown (Figure 6k). Collectively, these data demonstrate that STING signaling drives aging‐induced NAD^+^ decline in BMMs through IRF3‐ and P65‐mediated transcriptional activation of *Cd38*.

### 2.7 BMM‐Specific CD38 Inhibition from Middle Age Mitigates Chronic Inflammation and Bone Loss

The cGAS‐STING signaling pathway is increasingly recognized as a contributor to aging‐associated inflammation and neurodegeneration [9]. Additionally, CD38, reported to exhibit exclusive expression in M1 macrophages [31], mediates chronic inflammation across multiple metabolic tissues during aging [32]. We therefore investigated whether this pathway contributes to chronic inflammation in naturally aged bone. Notably, *Sting^ΔMφ^*‐18 M‐BMMs showed significantly reduced mRNA levels of *Cd38*, senescence markers (*Cdkn2a* and *Cdkn1a*), M1 macrophage markers (*Nos2*), and SASP‐related inflammatory cytokines (*Tnfa*, *Il1b*, and *Il6*) compared to WT‐18 M‐BMM controls. Conversely, M2 macrophage markers (*Arg1*, *Fizz1*, and *Ym1*) showed either increasing trends or remained unchanged (Figure 7a). The elevated mRNA levels of M1 macrophage markers (*Nos2*) and inflammatory cytokines (*Tnfa*, *Il1b*, and *Il6*) in 18 M‐BMMs paralleled the increases observed in bone marrow, indicating that 18 M‐BMMs predominantly undergo pro‐inflammatory M1‐like polarization (Figure S7a). Moreover, the increased mRNA levels of inflammatory cytokines (*Tnfa*, *Il1b*, and *Il6*) in 3 M‐BMMs treated with SN‐rDPP4 or co‐cultured with 18 M‐BMAds were reduced following *Cgas* or *Sting* knockdown, underscoring the crucial role of BMM STING signaling activation in driving chronic inflammation during skeletal aging (Figure S7b). Critically, CD38 inhibitor 78c effectively suppressed SN‐rDPP4‐ or 18 M‐BMAds‐induced inflammatory responses in 3 M‐BMMs (Figure S7c), collectively establishing the cGAS‐STING‐CD38 axis as a central regulator of skeletal aging‐associated inflammation.

**FIGURE 7:**
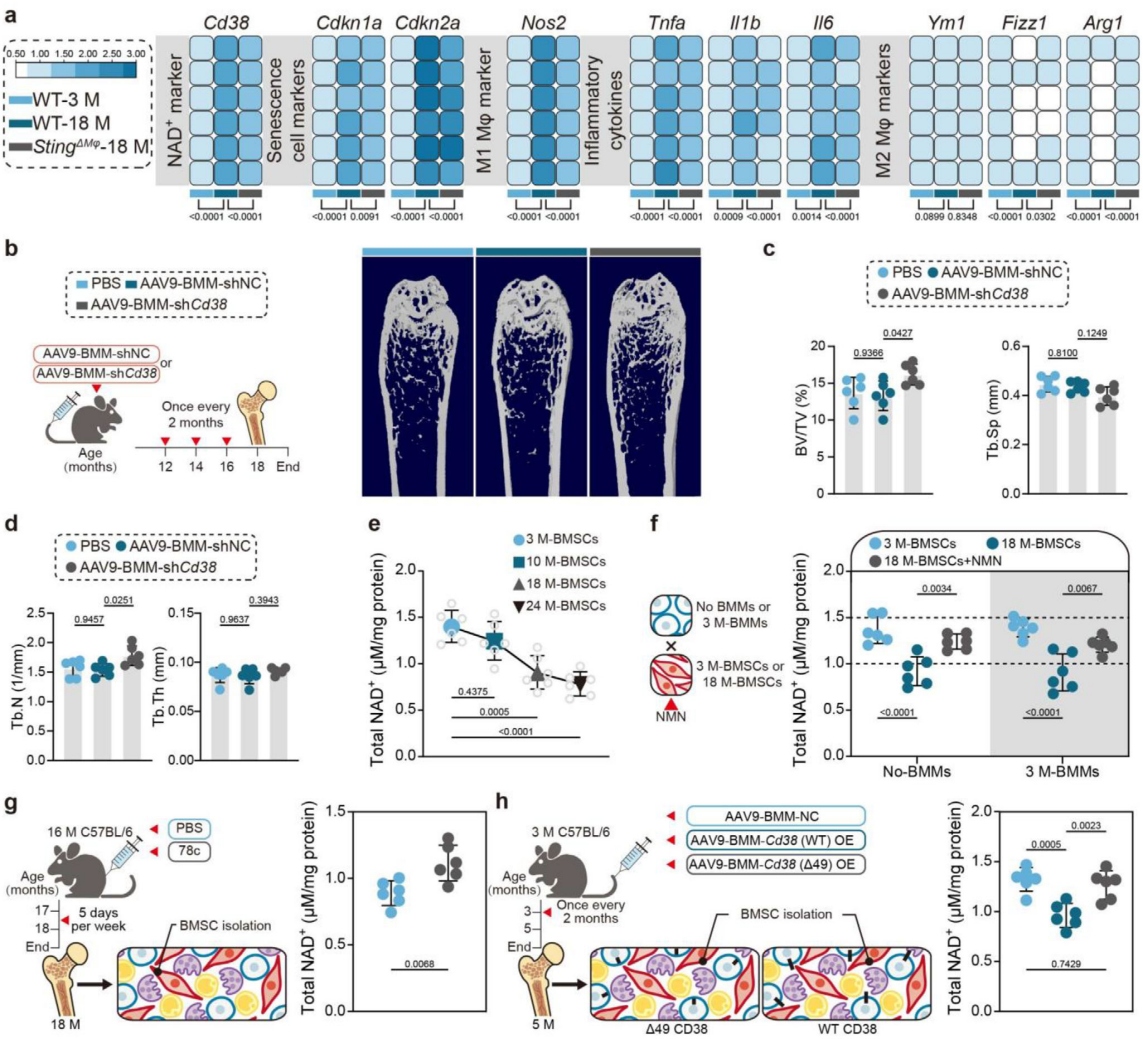
Enhanced CD38 ecto‐NADase activity in BMMs during skeletal aging impairs NMN availability to BMSCs. (a) qPCR analysis of *Cd38*, *Cdkn2a*, *Cdkn1a*, *Nos2*, *Tnfα*, *IL1b*, *IL6*, *Ym1*, *Fizz1*, and *Arg1* mRNA levels in bone marrow from 3‐ or 18‐month‐old WT and 18‐month‐old *Sting^ΔMφ^* mice (*n* = 6 biological replicates). (b—d) Representative micro‐CT images (b) and quantitative analysis of BV/TV, Tb. N, Tb.Th, and Tb.Sp (c,d) from AAV9‐BMM‐shNC‐treated or AAV9‐BMM‐sh*Cd38*‐treated male mice (*n* = 6 biological replicates). (e) WST‐8 analysis of NAD^+^ levels in 3, 10, 18, and 24 M‐BMSCs (*n* = 6 biological replicates). (f) WST‐8 analysis of NAD^+^ levels in BMSCs under co‐culture conditions (*n* = 6 biological replicates). (g) WST‐8 analysis of NAD^+^ levels in BMSCs from the bone marrow of mice treated with PBS or 78c from 16‐ to 18‐month‐old (*n* = 6 biological replicates). (h) WST‐8 analysis of NAD^+^ levels in BMSCs from the bone marrow of mice injected with AAV9 for BMM‐specific overexpression of WT or Δ49 mouse *Cd38* (*n* = 6 biological replicates). Data are shown as mean ± SD.

Interestingly, prior studies documented that 3‐month‐old *Cd38^−/−^* mice exhibit reduced bone mineral density at all examined skeletal sites (lumbar vertebrae L4‐L6, tibia, and femur) compared to WT littermates. However, no significant differences in bone mineral density were observed between 5‐month‐old *Cd38^−/−^* mice and their WT littermates at any site, with even a non‐significant increasing trend noted [33]. This pattern resembles the age‐dependent bone loss phenotype we observed in *Sting^ΔMφ^* mice, suggesting that CD38 may differentially regulate bone metabolism during developmental versus advanced aging stages, with a potential role in promoting bone loss from middle age onward. To validate this hypothesis, AAV9‐mediated BMM‐specific *Cd38* knockdown (AAV9‐BMM‐sh*Cd38*) was implemented in 12‐ to 18‐month‐old mice (Figure 7b, Figure S7d). Compared to age‐matched controls, BMM‐specific *Cd38* knockdown delayed bone loss, as evidenced by higher BMD, BV/TV, and Tb. N, along with reduced Tb.Sp (Figure 7b–d, Figure S7e). Mechanistically, BMM‐specific *Cd38* knockdown significantly reduced mRNA levels of senescence markers, M1 macrophage markers, and SASP‐related inflammatory cytokines in bone marrow (Figure S7f). Concurrently, elevated NAD^+^ levels were detected in the bone marrow microenvironment (Figure S7g), indicating that increased NAD^+^ bioavailability correlates with delayed skeletal aging progression.

### 2.8 CD38 Ecto‐NADase Activity in BMMs Contributes to NAD^+^ Decline in BMSCs

Given the established role of increased CD38 in BMMs in regulating chronic inflammation and NAD^+^ depletion, coupled with the observation that CD38 inhibition mitigates bone loss, the presence of additional regulatory mechanisms was hypothesized. CD38 exhibits a unique type II membrane topology with its catalytic domain oriented extracellularly, thus not only depleting intracellular NAD^+^ through intrinsic NADase activity but also regulating the availability of the NAD^+^ precursor NMN to other cells through its ecto‐NADase function in metabolic tissues including visceral white adipose tissue and liver [31, 34, 35]. Since the activation of the cGAS‐STING‐CD38 pathway appears to be primarily localized to BMMs, we next investigated whether BMM CD38 influences NAD^+^ homeostasis in BMSCs. Given the central role of BMSCs in bone formation, we measured NAD^+^ levels in BMSCs from 3‐ to 24‐month‐old mice (3, 10, 18, and 24 M‐BMSCs) and observed a progressive decline (Figure 7e). This finding raised the possibility that the age‐associated reduction of NAD^+^ in BMSCs may be linked to enhanced CD38 ecto‐NADase activity in BMMs. Consistent with this idea, the NMN transporter Slc12a8 [36] was expressed in BMSCs, suggesting their capacity to take up extracellular NMN to support intracellular NAD^+^ salvage biosynthesis (Figure S7h). To further test this possibility under aged skeletal conditions, we found that NMN supplementation effectively restored the decreased NAD^+^ levels in 18 M‐BMSCs cultured alone or with 3 M‐BMMs (Figure 7f).

For in vivo validation, 16‐month‐old mice treated with CD38 inhibitor 78c for two consecutive months exhibited partial NAD^+^ restoration in BMSCs (Figure 7g). Furthermore, AAV9 vectors expressing either full‐length CD38 (AAV9‐BMM‐*Cd38* (WT) OE) or an intracellularly truncated CD38 mutant (AAV9‐BMM‐*Cd38* (Δ49) OE) under the *F4/80* promoter were used to achieve conditional CD38 overexpression in 3‐month‐old mice. While WT‐CD38 predominantly localizes to the plasma membrane, the Δ49 mutant remains entirely intracellular [34]. Critically, BMM‐specific overexpression of WT‐CD38, but not Δ49‐CD38, reduced NAD^+^ levels in BMSCs, confirming that CD38 ecto‐NADase activity drives aging‐associated NAD^+^ decline in BMSCs (Figure 7h).

### 2.9 Osteogenic Differentiation Impairment in BMSCs by the DPP4‐STING‐CD38 Axis

The STING signaling‐mediated CD38 expression in BMMs suggests potential involvement in regulating NAD^+^ homeostasis in BMSCs. Accordingly, knockdown of *cgas* or *Sting* reversed the NAD^+^ decline in 18 M‐BMSCs induced by 3 M‐BMMs treated with SN‐rDPP4 or co‐cultured with 18 M‐BMAds (Figure S8a,b). In contrast, compared to WT‐18 M‐BMMs, *Sting^ΔMφ^*‐18 M‐BMMs also failed to affect NMN‐induced NAD^+^ increases in 18 M‐BMSCs (Figure S8c,d). Moreover, in vivo analysis revealed that *Sting^ΔMφ^*‐18 M mice exhibited not only significantly reduced *Cd38* mRNA expression (Figure 7a) but also higher NAD^+^ levels in bone marrow compared to WT mice (Figure S8e). Critically, BMM‐specific overexpression of WT‐CD38, but not Δ49‐CD38, abolished the elevated NAD^+^ levels in BMSCs of *Sting^ΔMφ^*‐18 M mice (Figure S8f,g). These results demonstrate that STING signaling in BMMs during aging regulates NAD^+^ decline in BMSCs through CD38 ecto‐NADase activity.

Intracellular NAD^+^ decline‐mediated mitochondrial dysfunction and apoptosis are associated with the development of various diseases. The potential induction of mitochondrial dysfunction and impaired osteogenic differentiation in BMSCs via BMM STING‐CD38 signaling‐mediated NAD^+^ decline was examined. Compared to 3 M‐BMSCs, 18 M‐BMSCs exhibited lower mitochondrial membrane potential (Figure S9a), reduced ATP production (Figure S9b), and elevated expression of the apoptosis marker caspase‐3 (Figure S9c). NMN supplementation effectively restored mitochondrial function in 18 M‐BMSCs when cultured alone or with 3 M‐BMMs (Figure S9a–c). However, replacing 3 M‐BMMs with 18 M‐BMMs abolished this restoration of mitochondrial function in 18 M‐BMSCs (Figure S9d–f). Similarly, diABZI‐treated, SN‐rDPP4‐treated, or 18 M‐BMAds‐co‐cultured 3 M‐BMMs replicated the suppression (Figure S9d–f). These effects were reversed by *cgas*/*Sting* knockdown in 3 M‐BMMs (Figure S9g–o). Furthermore, compared to 18 M‐BMSCs from WT mice (WT‐18 M‐BMSCs), those from *Sting^ΔMφ^* mice (*Sting^ΔMφ^*‐18 M‐BMSCs) displayed higher mitochondrial membrane potential (Figure 8a), more stable mitochondrial permeability transition pore (mPTP) opening (Figure 8b), elevated basal and maximal respiratory oxygen consumption rate (OCR) (Figure 8c), increased ATP production (Figure S10a), and reduced caspase‐3 expression (Figure S10b). Under osteogenic differentiation conditions, enhanced ALP activity and increased mineralized nodule formation (Figure 8d) indicated improved differentiation capacity. The expression of osteogenesis‐related proteins RUNX2 and BMP2 also showed an increasing trend, although this did not reach statistical significance (Figure 8e, Figure S10c). Because these BMSCs were assayed ex vivo in the absence of BMMs, the functional improvements observed in *Sting^ΔMφ^*‐18 M‐BMSCs suggest that BMM STING signaling exerts chronic detrimental effects on BMSC function in vivo, representing an extrinsic influence superimposed upon the intrinsic aging program of BMSCs. Analogously, BMSCs from 18‐month‐old mice with BMM‐specific *Cd38* knockdown also showed improved mitochondrial function (Figure 8f–h, Figure S10d,e) and osteogenic differentiation capacity (Figure 8i,j, Figure S10f) compared to those from control mice. In summary, the STING‐CD38 signaling axis in BMMs during skeletal aging triggers NAD^+^ decline in BMSCs, consequently inducing mitochondrial dysfunction and apoptosis, ultimately impairing bone formation.

**FIGURE 8:**
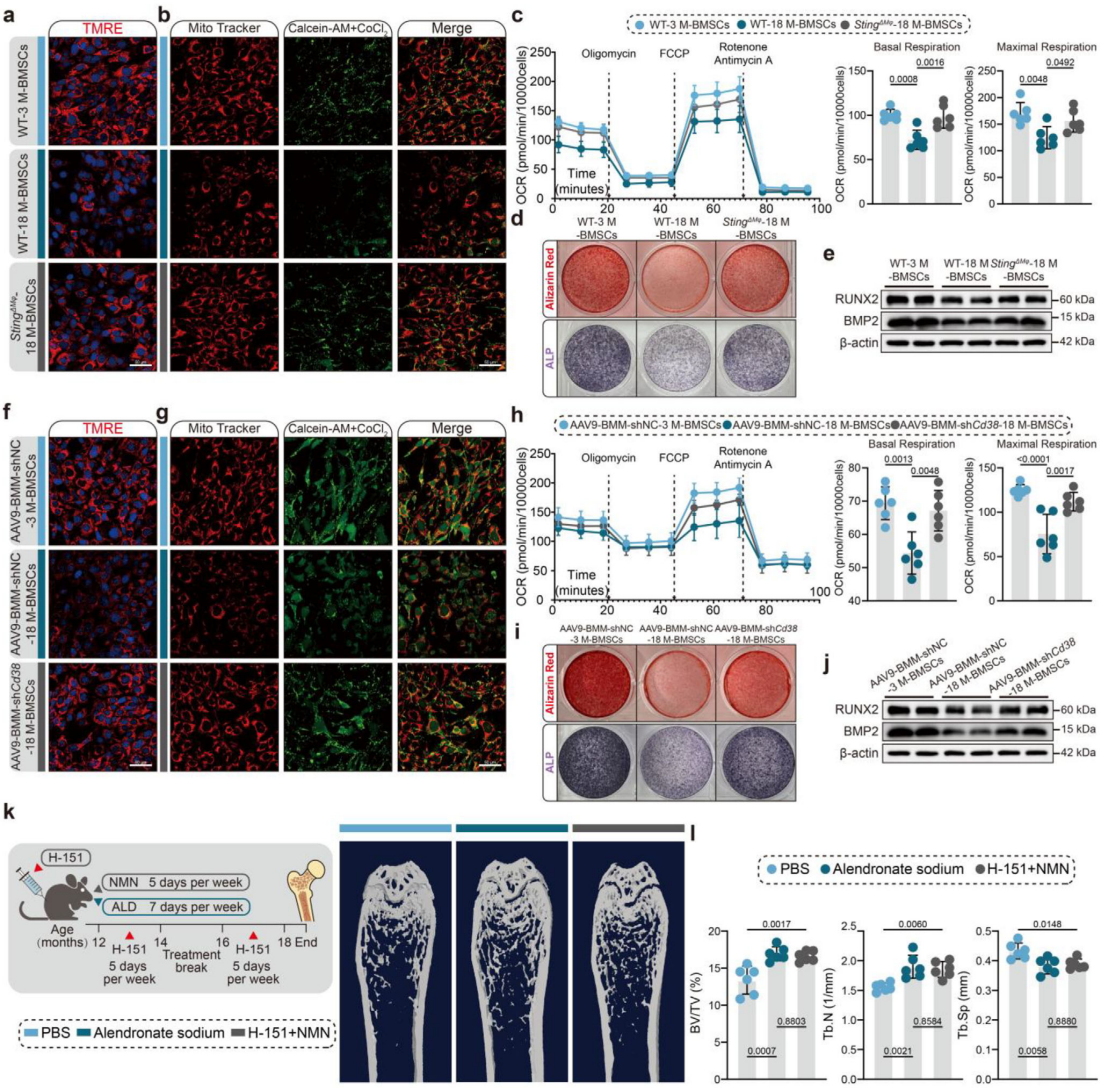
Combined pharmacological inhibition of STING and NMN supplementation enhances osteogenic differentiation of BMSCs to mitigate age‐related bone loss. (a) Representative images of tetramethylrhodamine ethyl ester (TMRE) staining of mitochondrial membrane potential in WT‐3 M‐/WT‐18 M‐BMSCs and *Sting^ΔMφ^*‐18 M‐BMSCs (scale bar: 50 µm). (b) Representative images of Calcein‐AM (green) and MitoTracker (red) staining for mitochondrial permeability transition pore opening in WT‐3 M‐/WT‐18 M‐BMSCs and *Sting^ΔMφ^*‐18 M‐BMSCs (scale bar: 50 µm). (c) Seahorse analysis of OCR in WT‐3 M‐/WT‐18 M‐BMSCs and *Sting^ΔMφ^*‐18 M‐BMSCs, with quantitative analysis of basal and maximal respiration levels (*n* = 6 biological replicates). (d) Representative images of Alizarin Red (red) and ALP (purple) staining for osteogenic differentiation activity in WT‐3 M‐/WT‐18 M‐BMSCs and *Sting^ΔMφ^*‐18 M‐BMSCs. (e) Representative immunoblots of RUN2 and BMP2 protein levels in WT‐3 M‐/WT‐18 M‐BMSCs and *Sting^ΔMφ^*‐18 M‐BMSCs (*n* = 3 biological replicates). (f) Representative images of TMRE staining of mitochondrial membrane potential in BMSCs from 3‐ or 18‐month‐old AAV9‐BMM‐shNC‐treated or 18‐month‐old AAV9‐BMM‐sh*Cd38*‐treated mice (scale bar: 50 µm). (g) Representative images of Calcein‐AM (green) and MitoTracker (red) staining for mitochondrial permeability transition pore opening in BMSCs from 3‐ or 18‐month‐old AAV9‐BMM‐shNC‐treated or 18‐month‐old AAV9‐BMM‐sh*Cd38*‐treated mice (scale bar: 50 µm). (h) OCR analysis in BMSCs from AAV9‐BMM‐shNC‐treated 3 M‐BMSCs or AAV9‐BMM‐sh*Cd38*‐treated 3 M‐/18 M‐BMSCs, with quantitative analysis of basal and maximal respiration levels (*n* = 6 biological replicates). (i) Representative images of Alizarin Red (red) and ALP (purple) staining for osteogenic differentiation activity in BMSCs from 3‐ or 18‐month‐old AAV9‐BMM‐shNC‐treated or 18‐month‐old AAV9‐BMM‐sh*Cd38*‐treated mice. (j) Representative immunoblots of RUNX2 and BMP2 protein levels in BMSCs from 3‐ or 18‐month‐old AAV9‐BMM‐shNC‐treated or 18‐month‐old AAV9‐BMM‐sh*Cd38*‐treated mice (*n* = 3 biological replicates). (k, l) Representative micro‐CT images (k) and quantitative analysis of BV/TV, Tb. N, and Tb.Sp (l) from PBS‐treated, ALD‐treated, or H‐151 and NMN co‐treated mice (*n* = 6 biological replicates). Data are shown as mean ± SD.

### 2.10 Combined Pharmacological Inhibition of STING and NMN Supplementation Initiated From Middle Age Onward Confers Potential Bone‐Protective Effects

Given that BMM STING signaling serves as a direct nexus connecting BMAT accumulation, low‐grade inflammation, and impaired bone formation, while enhancing downstream CD38 activity to deplete NAD^+^, pharmacological inhibition of STING activation combined with NMN supplementation may mitigate bone loss by ameliorating the pathological marrow microenvironment. To evaluate bone phenotype changes, the selective and well‐tolerated STING inhibitor H‐151 [37] was administered with continuous oral NMN supplementation. Consequently, treated mice exhibited significantly increased BMD, BV/TV, and Tb. N, along with significantly lower Tb.Sp, demonstrating anti‐osteoporotic efficacy comparable to the clinical first‐line drug alendronate sodium (ALD) (Figure 8k,l, Figure S11a). Calcein double‐labeling assays revealed elevated MAR in femora (Figure S11b). Simultaneously, H‐151 and NMN co‐treatment downregulated mRNA levels of senescence markers, M1 macrophage markers, and SASP‐related inflammatory cytokines in bone marrow (Figure S11c), while improving mitochondrial function and increasing NAD^+^ levels in BMSCs (Figure S11d–i). Collectively, these data demonstrate that pharmacological inhibition of STING contributes to comprehensive management of the complex aged bone marrow microenvironment, thereby delaying bone loss.

## 3 Discussion

The intricate interplay among BMAT expansion, low‐grade inflammation, and diminished bone turnover in the aged marrow microenvironment presents persistent challenges for osteoporosis interventions. Consequently, there is an urgent need to disrupt key signaling hubs that integrate these pathological features to enable comprehensive management of the aged bone marrow microenvironment. Here, STING signaling, which becomes prominently activated in BMMs during skeletal aging, was identified as a critical regulatory nexus. Notably, BMM‐specific STING deletion initiated from middle age conferred bone‐protective effects distinct from development and maturation, attributable to global alterations in the bone marrow microenvironment. Mechanistically, senescent BMAd‐derived sDPP4 functioned as an extracellular ligand for TLR4 on BMMs, while cooperatively acting with accumulated intracellular mtDNA to co‐activate STING signaling. Furthermore, activated STING signaling directly upregulated CD38 expression through downstream transcription factors NF‐κB and IRF3. Enhanced CD38 expression not only accelerates bone marrow inflammation but also, via its elevated ecto‐NADase activity, impairs NMN availability to BMSCs, leading to mitochondrial dysfunction and compromised osteogenic differentiation in these cells. Therapeutically, combined pharmacological STING inhibition and NMN supplementation from middle age onward demonstrated potent anti‐osteoporotic efficacy, thereby offering a strategy to resolve the aged bone marrow pathology.

While multiple impaired homeostatic mechanisms independently contribute to aging processes, many converge to generate a pro‐inflammatory state that collectively drives age‐related functional decline, with cGAS‐STING signaling recognized as pivotal in establishing this inflammatory condition [9, 38]. Importantly, the ability of type I interferon signaling to negatively regulate RANKL‐mediated osteoclast differentiation depends on STING [39]. This suggests that, unlike in other tissues, STING activation in bone may trigger two opposing processes. One involves enhanced bone resorption through NF‐κB activation, increased SASP factors, and elevated osteoclast formation coupled with reduced osteoblast numbers [40, 41], while the other is attenuated bone resorption resulting from STING‐dependent IFN‐I production that restricts osteoclast differentiation. Mice with constitutive STING activity develop reduced trabecular bone volume and thickness in caudal vertebrae at 6‐month‐old, accompanied by increased macrophage and monocyte numbers leading to systemic cytokine storm, though without altered osteoclast numbers [42]. These findings indicate that excessive STING activation preferentially promotes processes accelerating osteoporosis progression. To further clarify the role of STING in age‐related osteoporosis and enable targeted therapeutic strategies, its expression was examined across common bone marrow cell populations. Specifically, basal *STING* expression analysis revealed macrophage‐specific enrichment with progressive age‐dependent alterations. This expression pattern parallels the macrophage‐enriched STING signaling observed both in acute inflammatory conditions (such as myocardial infarction [43] and kidney injury [44]) and chronic inflammatory states (including benign airway stenosis fibrosis [45] and atherosclerosis [46]), reinforcing its central regulatory position within the immunometabolic network governing skeletal aging progression and therapeutic response.

In contrast to the marked inflammatory improvement observed with STING ablation in macrophages in the aforementioned studies, *Sting^ΔMφ^* male mice exhibited skeletal alterations that diverged from anticipated bone mass enhancement. Specifically, from 3‐month‐old to approximately 10‐month‐old stages, bone mass in *Sting^ΔMφ^* mice was reduced compared to WT controls, similar to findings in another study [13] where 15‐month‐old *Sting^−/−^* male mice or 2‐month‐old *Sting^ΔMφ^* female mice were examined 2 months post‐ovariectomy. Notably, this pattern reversed from approximately 18 months onward, with *Sting^ΔMφ^* mice displaying higher femoral bone mass than WT, although this increase at the final 24‐month observation point lacked statistical significance. Critically, progressive attenuation of bone loss was evident from around 10 months onward, culminating in eventual phenotypic reversal by 24 months. This aligns with observations in a previous study [42] where 16‐ to 18‐month‐old *Sting^−/−^* mice showed trending increases in caudal vertebral trabecular bone volume accompanied by significantly increased trabecular number. While cortical bone volume, cross‐sectional thickness, and bone area were significantly reduced in caudal vertebrae, this cortical reduction might reflect load‐sharing redistribution resulting from sufficient mechanical support provided by increased trabecular bone. From 12 to 18 months of age, BMM‐specific STING knockdown was performed in normal mice, which validated a key observation: unlike during bone development, where STING deficiency accelerates bone resorption by impairing IFN‐I‐mediated restraint of osteoclast differentiation, STING inhibition in BMMs from middle age onward attenuates bone loss. This finding confirms that the role of BMM STING in bone homeostasis shifts with age, favoring bone preservation when inhibited in later life stages. A plausible explanation is that given the predominant influence of complex interactions within the bone marrow microenvironment on bone mass during advanced aging, the effects of BMM STING activation should be evaluated under more physiologically integrated conditions. For instance, in co‐culture systems with BMSCs or vascular endothelial cells, BMM STING deficiency demonstrated effects beyond promoting osteoclast differentiation by restoring impaired bone formation and angiogenesis in other cell types. Consequently, the net effect from middle age reflects compensatory masking of STING ablation‐induced bone loss through restored formation. These findings collectively indicate that STING signaling in bone marrow macrophages exerts stage‐specific regulation of skeletal homeostasis, demonstrating detrimental effects only during aging progression. Notably, this observation is consistent with recent studies across diverse pathological contexts, including cancer [47], musculoskeletal disorders [41], and diabetic kidney disease [48], highlighting the context‑dependent dual effects of STING signaling.

The positive correlation between BMAT expansion and bone loss has been widely established. Specifically, BMATs actively respond to physiological and environmental stimuli such as aging, diabetes, ovariectomy, and glucocorticoid treatment, to become spatially proximate to other marrow cell populations. Thereby, through secreted adipokines, they impair physiological functions and contribute to bone loss [49, 50, 51, 52]. Notably, specific depletion of BMAT [53], inhibition of lipid secretion [54], or SASP factor [5] blockade partially delays aging in skeletal and/or vascular systems. In this study, continuous administration of 18 M‐BMAds‐CM for four weeks induced sustained activation of STING, likely reflecting the cumulative effects of BMAd‐derived factors within the bone marrow microenvironment. Building on these findings, we demonstrate for the first time that BMAd‐derived sDPP4 acts as a key SASP factor in activating the innate immune system. DPP4, a ubiquitously expressed serine protease, specifically cleaves off N‐terminal dipeptides from proteins with proline or alanine at the second amino acid position, thereby regulating the activity of numerous chemokines and peptide hormones [55]. Before its identification as a novel adipokine, DPP4 was primarily targeted for type 2 diabetes treatment due to its ability to degrade incretin hormones, principally glucagon‐like peptide 1. Interestingly, multiple studies have reported unexpected bone‐protective effects of common DPP4 inhibitors such as sitagliptin [56], saxagliptin [57], and vildagliptin [58] in clinical and preclinical models, suggesting DPP4 involvement in bone remodeling. Consistently, elevated *Dpp4* expression occurs in aged mouse distal tibiae [19]; similarly, sDPP4 levels are substantially increased in aged femoral marrow supernatants, predominantly originating from senescent BMAds. Critically, the results showed that rDPP4 treatment significantly activated BMM STING signaling in mouse bone marrow. Although all injected groups, including PBS controls, underwent identical intramedullary procedures and therefore shared potential procedure‐related effects, repeated intramedullary injection may nevertheless introduce local mechanical trauma and transient inflammation that could influence the bone marrow microenvironment and bone remodeling. Contralateral limbs and local injury‐associated markers were not assessed in the present study and should be considered in future investigations. Since rDPP4 exposure moderately impairs osteogenic differentiation without affecting adipogenic differentiation [19], BMAd‐derived sDPP4 may additionally accelerate bone loss through indirect interactions with BMMs. Notably, DPP4 also possesses intrinsic serine protease activity capable of processing a broad range of substrates, yet we did not formally evaluate a catalytically inactive DPP4 mutant in the present study. Therefore, whether the observed bone phenotypes require, or are modulated by, DPP4 catalytic activity remains to be determined in future investigations. Moreover, combining rDPP4 with aged bone marrow supernatant, rather than rDPP4 alone, synergistically enhanced STING activation, accompanied by TLR4 upregulation. This effect potentially relates to the higher endotoxin levels in aged bone marrow supernatant. Meanwhile, TLR4‐mediated STING activation also depended on age‐associated mtDNA accumulation in BMMs, and mtDNA depletion only partially reduced p‐STING levels (by approximately 40–50%), indicating that prominent BMM STING activation from middle age onward results from the combined effects of BMAT expansion, endotoxin elevation, and mitochondrial stress convergence. This cooperative activation model further explains why direct targeting of STING, as a central signaling convergence node, yields more pronounced therapeutic effects than targeting a single upstream factor such as sDPP4, thereby mechanistically supporting the role of STING as a key hub in aging‑associated skeletal pathology.

CD38, broadly expressed in immune cells, exhibits inflammatory condition‐dependent upregulation [59]. Beyond its established role as an M1 macrophage marker, CD38 functions as a pivotal NAD^+^ salvage enzyme regulating NAD^+^ homeostasis during macrophage polarization [29]. Studies demonstrate that in aged metabolic tissues, senescent cell‐derived SASP factors induce robust CD38 expression in macrophages [31]. Through its unique extracellular catalytic domain orientation, CD38 degrades extracellular NMN and NAD^+^, thereby limiting precursor availability for neighboring cells [31, 34]. Consistently, while only 1% of BMMs expressed CD38 in 3‐month‐old mouse femoral bone marrow, over 80% of BMMs highly expressed CD38 in 18‐month‐old mice [6]. Besides, CD38‐dependent NAD^+^ salvage pathway activation coincided with BMM STING signaling, with CD38 expression directly correlating with BMAd‐derived sDPP4‐mediated STING activation. Importantly, high CD38 expression in BMMs not only amplifies the inflammatory milieu but also hijacks key NAD^+^ salvage precursors by depleting NMN in the bone marrow microenvironment, ultimately leading to mitochondrial dysfunction and impaired osteogenic differentiation in neighboring BMSCs. Intriguingly, *Cd38^−/−^* mice mirrored *Sting^−/−^* phenotypes: enhanced osteoclast activity at the 3‐month‐old stage yet reversed the significant bone loss observed at 3 months by the 5‐month‐old stage, even displaying an increasing trend [33]. This trajectory aligned with the characteristic rapid‐decline‐then‐attenuation pattern in *Sting^ΔMφ^* mice. Furthermore, BMM‐specific *Cd38* knockdown from the 12‐ to 18‐month‐old stages also produced bone‐protective effects analogous to *Sting* knockdown, highlighting the dual and stage‐specific role of CD38 in skeletal aging that depends on comprehensive alterations in the bone marrow microenvironment. Given the central role of BMM STING‐CD38 signaling in promoting bone loss from middle age, combined STING inhibitor (H‐151) and NMN supplementation were administered. Significantly, this regimen delayed bone loss while favorably modulating inflammation, senescence markers, and NAD^+^ levels in bone marrow, demonstrating the translational potential for age‐related osteoporosis. As this proof‐of‐concept study was designed to assess the combination strategy, monotherapy treatment arms were not included. Therefore, a formal evaluation of the individual versus combined effects of these interventions warrants future investigation.

Several limitations of this study should be acknowledged. First, while the *Lyz2*‐Cre mouse model effectively targets BMMs, it also induces *Sting* deletion in other myeloid cells, including osteoclast precursors. Although we corroborated BMM‐specific functions using AAV9‐BMM‐sh*Sting* under the *F4/80* promoter, potential contributions from other myeloid cells such as neutrophils cannot be completely excluded. Therefore, the use of a more refined model in future studies—such as a tamoxifen‐inducible, BMM‐specific knockout system—would enable temporal control of STING deletion and facilitate more precise dissection of its stage‐specific roles in skeletal aging. Additionally, because the *Pdgfrα* promoter used in the AAV9 construct is not exclusively active in mature BMAds, potential contributions from PDGFRα⁺ stromal and adipogenic progenitor cells cannot be completely excluded. Furthermore, this study did not include early bone mass assessments at 1–2 months of age, and the observation intervals were relatively broad. Consequently, the role of BMM STING during developmental stages, as well as the optimal therapeutic window for targeted intervention, remains to be elucidated in future studies. Beyond sDPP4, multiple additional cytokines were identified within aged marrow supernatants. Accordingly, their synergistic interactions with sDPP4 in bone metabolism require comprehensive screening. Furthermore, investigating whether CD38‐mediated NMN depletion similarly affects osteoclast differentiation and function via modulation of the bone marrow microenvironment would help delineate the relative contributions of bone resorption versus bone formation to the bone‐protective effects observed with STING inhibition. Finally, although the combination of H‐151 and NMN supplementation demonstrates bone‐protective potential, the observed benefits of this systemic pharmacological intervention may reflect a combination of local bone marrow effects and systemic improvements in metabolic or inflammatory status. Future studies incorporating local delivery approaches or cell‐type‐specific targeting strategies will be essential to dissect the relative contributions of local versus systemic effects.

In summary, this work establishes that BMAT expansion, innate immune‐mediated low‐grade inflammation, and impaired bone formation, three hallmark pathological features of skeletal aging, are interconnected through a defined molecular pathway. Consequently, these findings position STING signaling in bone marrow macrophages as a pivotal integration hub, highlighting its targeting potential for comprehensive management of the complex bone marrow microenvironment during skeletal aging.

## 4 Methods

### 4.1 Animal Models

*Sting^flox/flox^* mice (S‐CKO‐15494) and *Lyz2*‐Cre mice (C001003) were sourced from Cyagen Biosciences Inc. (Suzhou, China). Using CRISPR‐Cas9 technology, *Sting^flox/flox^* mice were generated on a C57BL/6 background, with two *loxP* sequences inserted in *Sting1* intron 5 and downstream of the stop codon. Subsequently, *Sting^flox/flox^* mice were crossed with *Lyz2*‐Cre mice to produce male *Sting^flox/flox^*; lyz2‐Cre^+^ mice (*Sting^ΔM^*) and *Sting^flox/flox^*; *Lyz2*‐Cre^−^ littermates (WT controls).‌Additionally, wild‐type C57BL/6J male mice were purchased from Vital River Laboratory Animal Technology (Beijing, China). All mice were maintained under specific pathogen‐free conditions with controlled temperature at 22°C–24°C with 12 h light/dark cycles, receiving standard chow and water ad libitum. Throughout experiments and analyses, investigators were blinded to treatment/genotype groups, while group randomization was performed using a random number generator. All animal experimental protocols were reviewed and approved by the Institutional Animal Care and Use Committee of Shandong University of Traditional Chinese Medicine (SDUTCM20240321001).

### 4.2 In Vivo Experiments

For BMM‐specific knockdown of *Sting* or *Cd38*, AAV9 vectors carrying short hairpin RNA targeting *Sting1* or *Cd38* under *F4/80* as the promoter (AAV9‐BMM‐sh*Sting* or AAV9‐BMM‐sh*Cd38*), along with their control vectors (AAV9‐BMM‐shNC), were constructed by GENECHEM (Shanghai, China). Similarly, for BMAd‐targeted *Dpp4* knockdown, AAV9 vectors carrying short hairpin RNA targeting *Dpp4* with *Pdgfra* as the promoter (AAV9‐BMAd‐sh*Dpp4*) and their control vector (AAV9‐BMAd‐shNC) were generated by GENECHEM (Shanghai, China). Regarding administration protocols, AAV9 virus was delivered via tail vein injection at a dose of 1 × 10^12^ vg/mouse. Mice received injections every 2 months from 12‐month‐old to 18‐month‐old (a total of 3 injections). To specifically overexpress WT CD38 or truncated CD38 (Δ49) in BMMs, AAV9 carrying vector genomes with *F4/80* as the promoter (AAV9‐BMM‐*Cd38* (WT) OE or AAV9‐BMM‐*Cd38* (Δ49) OE) and control vectors (AAV9‐BMM‐NC) were generated by GENECHEM (Shanghai, China). AAV9 was administered to mice through the tail vein at a dose of 1 × 10^12^ vg/mouse. Mice received a single injection from 3 months old to 5 months old.

In the LPS (Sigma, L5418) experiment, 3‐month‐old male C57BL/6J mice were intraperitoneally injected with LPS (0.25 mg/kg) or PBS twice weekly for 4 weeks. Conversely, rDPP4 (Sino Biological, 10688‐H08H) administration involved once‐weekly intramedullary injections (0.2 µg per injection) for 4 weeks. In parallel, 16‐month‐old male C57BL/6J mice received 78c (MedChemExpress, HY‐123999; 10 mg/kg) or PBS intraperitoneally 5 days per week for 8 weeks. In the intra‐bone marrow injection experiment of conditioned medium (CM), BMAds were isolated from the bilateral femurs and tibiae of 3‐month‐old or 18‐month‐old male C57BL/6J mice to prepare CM. Approximately 5 × 10^4^ BMAds were seeded in 48‐well plates and cultured in serum‐free α‐MEM for 24 h, after which the CM was collected. The CM was subsequently concentrated 10‐fold using centrifugal filter units with a 3 kDa molecular weight cutoff. Recipient 3‐month‐old male C57BL/6J mice were anesthetized, and a small hole was drilled into the patellar groove of the right distal femur using a sterile 27G needle. A microsyringe was then used to inject 10 µL of concentrated CM or PBS into the bone marrow cavity once weekly for 4 weeks. In the H‐151 (MedChemExpress, HY‐112693) and NMN (BONTAC, China) experiments, 12‐month‐old male C57BL/6J mice were intraperitoneally injected with H‐151 (5 mg/kg) or PBS 5 days per week for 8 weeks. After an 8‐week treatment break, the treatment was resumed for another 8 weeks until the mice reached 18 months of age. Concurrently, NMN (200 mg/kg/day) was administered orally 5 days weekly across 6 months.

### 4.3 Cell Culture

BMMs were isolated following established methodology [6]. Specifically, femora and tibiae were collected from mice, with both ends excised to fully expose the marrow cavity. Subsequently, bone marrow was flushed using ice‐cold PBS through repeated irrigation cycles. The resulting crude suspension was passed through a 70 µm cell strainer, then centrifuged with supernatant removal. Red blood cell lysis buffer was added and incubated at room temperature for 5 min, followed by another centrifugation step and supernatant removal. The pellet was resuspended in PBS to obtain a single‐cell suspension. After incubation with Anti‐Mouse CD16/32 (Elabscience, E‐AB‐F0997A) for 10 min, cells were stained with CD45‐APC‐Cy7 (BioLegend, 103116), CD11b‐BB700 (BD Horizon, 566417), and F4/80‐FITC (BioLegend, 123108) for 20 min. Ultimately, CD45^+^ CD11b^+^ F4/80^+^ cells were purified as BMMs using a FACS Aria instrument (BD Biosciences, USA).

BMSCs were isolated according to published procedures [60]. Following a similar procedure to BMM isolation, after preparing the bone marrow single‐cell suspension, cells were incubated with Anti‐Mouse CD16/32 (Elabscience, E‐AB‐F0997A) for 10 min. Thereafter, staining was conducted with Sca‐1‐PE (BioLegend, 108108), CD29‐FITC (BioLegend, 102206), CD45‐PerCP‐Cy5.5 (BioLegend, 103132), and CD11b‐APC‐Cy7 (BioLegend, 101226) for 20 min. Sca‐1^+^ CD29^+^ CD45^−^ CD11b^−^ cells were sorted as BMSCs using a FACS Aria instrument (BD Biosciences, USA).

BMAds were isolated as previously described [61]. Briefly, femora and tibiae were collected from mice, and both ends were removed to expose the bone marrow cavity. Next, bones were transferred to perforated‐bottom microcentrifuge tubes (0.6 mL), which were then inserted into larger microcentrifuge tubes (1.5 mL). Fresh bone marrow was collected by brief centrifugation. After red blood cell lysis buffer addition and 5‐min incubation at RT, samples were centrifuged at 3000 rpm for 5 min at RT. The floating adipocyte layer was collected and washed three times with PBS.

Pre‐osteoclasts and osteoclasts were generated by seeding isolated BMMs in 48‐well plates and inducing differentiation with osteoclastogenic medium containing 10 ng/ml M‐CSF (Novoprotein, CB34) and 50 ng/ml RANKL (Novoprotein, CJ94) for 3 or 6 days.

Osteoblasts were obtained by seeding BMMs in 48‐well plates and culturing them in osteogenic differentiation medium supplemented with 0.1 mm dexamethasone (Selleck, S1322), 10 mM β‐glycerol phosphate (Sigma, G5422), and 50 mm ascorbate‐2‐phosphate (MedChemExpress, HY‐103701) for 14 days.

Neutrophils were isolated using the Anti‐Ly‐6G MicroBeads UltraPure kit (Miltenyi Biotec, 130‐120‐337) via magnetic separation.

### 4.4 In Vitro Experiments

For targeted CD38 overexpression and knockdown of *Cgas*, *Sting*, *Irf3*, and *Rela* in BMMs, replication‐deficient adenoviral vectors encoding these constructs, including Ad‐*Cd38*‐oe, Ad‐sh*Cgas*, Ad‐sh*Sting*, Ad‐sh*Irf3*, Ad‐sh*Rela*, along with respective control vectors, were obtained from GENECHEM (Shanghai, China) and employed for cellular infection.

Regarding SN‐rDPP4 medium preparation, bone marrow supernatant was obtained following established protocols [6]. In brief, holes were drilled at both ends of the femora with a needle, and the bone marrow supernatant was collected by centrifugation. The entire procedure was performed under strict aseptic and pyrogen‐free conditions to prevent potential endotoxin contamination. The bone marrow supernatant was mixed with basal medium at a 1:10 ratio. Based on the highest reported sDPP4 levels in type 2 diabetic patients (approximately 600 ng/mL) [62], the basal medium containing bone marrow supernatant was supplemented with 500 ng/mL rDPP4 (Abcam, ab290094) to obtain the final SN‐rDPP4 medium.

Cell co‐culture experiments were performed using Transwell chambers (CORNING, USA). For BMAd‐BMM co‐culture studies, the following experimental designs were implemented: (1) BMAds from 3‐month‐old or 18‐month‐old male C57BL/6J mice were seeded in the upper chamber, while BMMs from 3‐month‐old male C57BL/6J mice were seeded in the lower chamber with 0.5 µM 78c (Selleck, S8960). After 48 h of co‐culture, BMMs were collected for subsequent analysis; (2) BMAds from 3‐month‐old male C57BL/6J mice were seeded in the upper chamber. BMMs from 3‐month‐old male C57BL/6J mice were seeded in the lower chamber and transfected with Ad‐shCgas or Ad‐shSting to knock down respective gene expression. Following 48 h of co‐culture, BMMs were collected for subsequent analysis.

In BMM‐bEnd.3 cell co‐culture experiments examining endothelial tube formation, BMMs from 3‐month‐old or 18‐month‐old male C57BL/6J mice were seeded in the upper chamber, while bEnd.3 cells (ATCC, USA) were maintained in the lower chamber. After 48 h of co‐culture, bEnd.3 cells were harvested and transferred onto Geltrex‐coated plates. Tube formation was assessed 6 h later.

For BMM‐BMSC co‐culture investigations, three experimental configurations were implemented: (1) In osteogenic differentiation assessments, BMMs from mice of different ages and genetic backgrounds were seeded in upper chambers, with BMSCs from 3‐month‐old male C57BL/6J mice in the lower chambers. BMSCs underwent continuous osteogenic differentiation induction. To minimize potential effects of differentiation medium on BMMs, Transwell chambers containing BMMs were replaced daily. Osteogenic differentiation of BMSCs was evaluated at days 7 and 14; (2) BMMs from 3‐month‐old male C57BL/6J mice transfected with Ad‐*Cd38* oe were seeded in upper chambers, whereas BMSCs from 3‐month‐old male C57BL/6J mice were seeded in lower chambers. After 24‐h co‐culture periods, BMSCs were collected for total NAD^+^ quantification; (3) BMMs from 3‐month‐old male C57BL/6J mice were seeded in upper chambers, while BMSCs from 3‐month‐old or 18‐month‐old male C57BL/6J mice were seeded in lower chambers with 100 µg/mL NMN. After 24 h of co‐culture, BMSCs were collected for total NAD^+^ quantification.

### 4.5 Micro‐CT Analysis

For trabecular bone microarchitecture assessment, left femora of mice were collected, fixed in 4% paraformaldehyde for 48 h, and then scanned using a Bruker Micro‐CT Skyscan 1276 system (Kontich, Belgium). The scanner parameters were set at a source voltage of 60 kV, a source current of 200 µA, an integration time of 205 ms, and a voxel size of 6.554883 µm. The region of interest for cancellous bone was defined as a segment starting 1 mm beneath the growth plate and extending 1 mm in length. Key morphometric parameters, including BV/TV, Tb. N, Tb.Th, and Tb. Sp, were quantified to characterize bone structural properties.

### 4.6 Bone Histomorphometry Analysis

Following collection, femoral samples were fixed in 4% paraformaldehyde for 24 h, then decalcified with 10% EDTA for 3 weeks, and finally embedded in paraffin. Using a microtome (Leica, Germany), 4 µm‐thick longitudinal bone sections were prepared and stained with H&E. The quantitative analysis of adipocyte number was performed with ImageJ. Calcein double‐labeling was used for dynamic histomorphometry analysis. The mice were intraperitoneally injected with calcein (20 mg/kg) 10 d and 3 d prior to euthanasia. The left femora of mice were collected, fixed in 4% paraformaldehyde for 2 d, and then dehydrated and embedded in methyl methacrylate. Non‐decalcified bone sections (10 µm) were prepared using a hard tissue microtome. All fluorescent images were acquired with a DMi8 fluorescence microscope (Leica, Germany), with MAR and MS/BS calculated via ImageJ analysis.

### 4.7 cGAMP Quantification Assay

cGAMP levels were measured by 2'3'‐cGAMP ELISA Kit (Cayman Chemical, 501700) according to the manufacturer's instructions. Prior to analysis, total protein from bone marrow was extracted with T‐PER Tissue Protein Extraction Reagent (Thermo Fisher, 78510), whereas cellular protein was isolated employing M‐PER Mammalian Protein Extraction Reagent (Thermo Fisher, 78503). Subsequently, each well received sequential additions of 100 µL of Immunoassay Buffer C, 50 µL of sample, 50 µL of 2'3'‐cGAMP‐HRP Tracer, and 50 µL of 2'3'‐cGAMP ELISA Polyclonal Antiserum. Following overnight incubation at 4°C, plates underwent five washing cycles using 300 µL of Wash Buffer per well. Thereafter, 175 µL of TMB Substrate Solution was added for chromogenic development, after which the enzymatic reaction was terminated with 75 µL of HRP Stop Solution per well. Absorbance was measured at 450 nm. The cGAMP concentration was normalized to the total protein concentration.

### 4.8 Cytosolic dsDNA Concentration Measurement

Cytosolic dsDNA was isolated using a Mitochondria Isolation Kit (Beyotime, C3601) according to the manufacturer's instructions. Briefly, bone marrow single‐cell suspensions or sorted BMMs were washed with ice‐cold PBS, then incubated in mitochondria isolation reagent at 4°C for 10 min. After homogenization, samples were centrifuged at 1000 *×g* for 10 min at 4°C. The supernatant, containing cytosolic components and mitochondria, was transferred to a new tube and further centrifuged at 12 000 *×g* for 15 min at 4°C to pellet the mitochondria. Residual mitochondrial or lysosomal contamination was removed using the Monarch PCR & DNA Cleanup Kit (New England Biolabs, T1030S). Cytosolic dsDNA concentration was measured using the PicoGreen dsDNA Assay Kit (Thermo Fisher, P7589). All procedures were conducted in accordance with the manufacturer's instructions.

### 4.9 Endotoxin Measurement

Serum and bone marrow endotoxin were determined with the Pierce Chromogenic Endotoxin Quantitation Kit (Thermo Fisher, A39552). Following dilution (1:10–1:100) under pyrogen‐free conditions, samples were heat‐inactivated at 70°C for 10 min, after which the collected samples were assayed following the standard kit protocol.

### 4.10 ALP and Alizarin RED Staining

BMSCs were induced to undergo osteogenic differentiation by culturing in α‐MEM supplemented with 10% fetal bovine serum, 0.1 mM dexamethasone, 10 mM β‐glycerol phosphate, and 50 mM ascorbate‐2‐phosphate for 7 or 14 days. On day 7, cells were stained with BCIP/NBT Alkaline Phosphatase Color Development Kit (Beyotime, C3206). On day 14, alizarin red staining was performed using the Alizarin Red S Solution (OriCell, ALIR‐10001), according to the manufacturer's instructions.

### 4.11 scRNA‐seq Analysis

The scRNA‐seq data of mouse femoral bone marrow were obtained from the GEO database (GSE145477). After quality control (excluding genes in < 3 cells, cells with < 200 genes, and doublets via Scrublet), the data were normalized and log‐transformed. We identified 4000 highly variable genes and regressed out technical covariates (total UMIs, mitochondrial, and ribosomal gene percentages). The data were scaled, and PCA was performed. Cell clustering was conducted using the Leiden algorithm on a neighborhood graph, with visualization in t‐SNE. Cell types were annotated based on marker genes from a published reference [1, 63]. *Tmem173* (*Sting1*) expression was visualized on t‐SNE and quantified across clusters via a dot plot.

### 4.12 Mass Spectrometry and 4D Label‐Free Quantitative Proteomics

Rat bone marrow supernatant was subjected to 4D label‐free quantitative proteomic analysis. Rat femora were selected because the volume of bone marrow supernatant obtainable from mouse femora by centrifugation was insufficient to meet the sample input requirements for 4D label‐free quantitative proteomics. The substantially greater supernatant volume provided by rat femora enabled robust protein identification and quantification while preserving the bone marrow microenvironment relevant to skeletal aging. Briefly, high‐abundance proteins were depleted using an affinity removal column. The resulting low‐abundance protein fraction was lysed using SDT buffer (4% SDS, 100 mm Tris‐HCl, pH 7.6) and quantified using a BCA assay. Thereafter, proteins were separated by SDS‐PAGE. In‐solution digestion was performed using the filter‐aided sample preparation method, involving reduction with DTT, alkylation with iodoacetamide, and overnight tryptic digestion. The resulting peptides were desalted and analyzed by LC‐MS/MS on an Orbitrap Exploris 480 mass spectrometer (Thermo Fisher, USA) coupled with an Easy nLC system (Thermo Fisher). Finally, MS data were processed using MaxQuant software (v.1.6.17.0) against the appropriate rat protein database. Protein quantification was based on normalized LFQ intensities, with differential expression defined as a fold change > 1.5 with *p* < 0.05 (Student's *t*‐test).

### 4.13 SPR Assay

The binding interaction between rTLR4 (Sino Biological, 10146‐H08B) and rDPP4 (Sino Biological, 10688‐H08H) was analyzed using a BIAcore T200 instrument (Cytiva, USA). Initially, rTLR4 was immobilized onto a CM5 sensor chip (Cytiva, USA) via the amine coupling method, involving EDC/NHS activation followed by ethanolamine blocking, achieving a final immobilization level of 8139.5 RU. rDPP4 was serially diluted two‐fold across a concentration range of 7.81–250 nm. Binding assays were performed at a flow rate of 30 µL/min with association and dissociation phases set to 120 s and 360 s, respectively. Finally, kinetic parameters were derived by globally fitting the sensorgrams to a 1:1 Langmuir binding model, as implemented in the BIAcore T200 evaluation software.

### 4.14 Co‐Immunoprecipitation

For protein‐protein interaction validation, HEK293T cells were transfected with the HA‐*Tlr4* plasmid or with various Flag‐*Dpp4* plasmids, including the full‐length, Δ47‐284, Δ285‐505, and Δ506‐760 truncation mutants, respectively. All plasmids were generated by GENECHEM (Shanghai, China). Following transfection, cells were lysed in immunoprecipitation (IP) lysis buffer (Beyotime, P0037) containing 1% protease inhibitor cocktail (Beyotime, P1005) at 4°C for 40 min. The resulting lysates were then centrifuged at 14 000 *×g* at 4°C for 15 min to obtain protein samples. Some protein samples were prepared as the Input control. The remaining protein sample was incubated with Anti‐Flag (MedChemExpress, HY‐K0207), Anti‐HA (MedChemExpress, HY‐K0201), and Anti‐IgG (Beyotime, P2171) magnetic beads at 4°C for 12 h. After five wash cycles with lysis buffer (each lasting 5 min at 4°C with continuous shaking), 40 µL of 1×loading buffer was added to each sample and heated at 98°C for 10 min to denature. The IP samples were analyzed by Western blotting.

### 4.15 Luciferase Reporter Assay

To investigate the transcriptional regulation of Cd38 by IRF3 or Rela, HEK293T cells were co‐transfected with the Cd38‐promoter luciferase reporter (*Cd38*‐luc, 500 ng), pRL‐*nTK* (a Renilla luciferase reporter; 20 ng), and either *Irf3* or *Rela* overexpression plasmids using Lipofectamine 3000 transfection reagent (Thermo Fisher, L3000‐015). All plasmids were generated by GENECHEM (Shanghai, China). After 48 h, cell lysates were prepared, and luciferase activities were measured with the Dual‐Luciferase Reporter Assay System (Promega, E1910). Specifically, firefly luciferase activity was normalized to the Renilla luciferase activity for data analysis.

### 4.16 ChIP Assay

ChIP was performed via the SimpleChIP Enzymatic Chromatin IP Kit (Cell Signaling Technology, 9003). BMMs from experimental groups were fixed with 1% formaldehyde, and the formaldehyde was then inactivated by the addition of glycine. Chromatin was digested with micrococcal nuclease and sonicated to produce fragments. Chromatin was immunoprecipitated by either control IgG, or IRF3 (Thermo Fisher, PA5‐88077) or NF‐κB p65 (Cell Signaling Technology, 8242) primary antibody. Following IP, the enriched DNA was reverse‐crosslinked and quantitatively analyzed by qPCR.

### 4.17 CD38 NADase Assay

CD38 NADase activity was determined following established methodology [64]. Briefly, cells were initially incubated in hydrolase activity buffer (0.25 M sucrose, 40 mM Tris pH 7.4) at RT for 15 min. This was followed by the addition of ε‐NAD (MedChemExpress, HY‐137592) for 1 h incubation at RT. Subsequently, fluorescence intensity was quantified at 300 nm excitation and 410 nm emission.

### 4.18 Mitochondrial Functional Assay

For the mitochondrial membrane potential assay, treated cells were stained with TMRE (Beyotime, C2001S) at 37°C for 20 min. Nuclei were then counterstained with DAPI for 2 min. All images were captured using a confocal laser scanning microscope (LSM 900, Zeiss, Germany).

For the mPTP opening assay, cells were first incubated with MitoTracker Red (Beyotime, C1032) at 37°C for 30 min to visualize mitochondria, then stained with calcein‐AM solution (Beyotime, C2009) at 37°C for 30 min. Subsequently, the staining solution was replaced with pre‐warmed culture medium containing 10% FBS, and the cells were further incubated at 37°C for 30 min under light‐protected conditions. Finally, nuclei were stained with DAPI for 2 min. All images were captured using a confocal laser scanning microscope (LSM 900, Zeiss, Germany).

The OCR was measured using a Seahorse XF96 Extracellular Flux Analyzer (Agilent, USA). BMSCs isolated from different age or treatment groups were seeded into XF 96‐well plates at a density of 10 000 cells per well. Following cell adherence, the culture medium was exchanged for XF base medium (supplemented with 10 mm glucose, 1 mM sodium pyruvate, and 2 mM L‐glutamine), with incubation at 37°C in a non‐CO_2_ environment for 1 h. OCR measurements were performed following sequential administration of mitochondrial modulators, including oligomycin (1 µm), carbonyl cyanide‐p‐trifluoromethoxyphenylhydrazone (FCCP, 0.5 µM), and a mixture of rotenone and antimycin A (1 µm each) under standard assay conditions.

Total NAD^+^ levels were measured using the NAD^+^/NADH Assay Kit (Beyotime, S0175). To minimize alterations in NAD^+^ levels, flow cytometric sorting of BMSCs was completed within 2 h, and all buffers were pre‑chilled. BMSCs were collected directly into ice‑cold PBS and immediately lysed using the kit‐provided buffer to prepare test samples. The samples were then incubated with alcohol dehydrogenase solution at 37°C for 10 min. Subsequently, chromogenic solution was added, followed by an additional 20‐min incubation at 37°C. Finally, the absorbance of the final reaction mixture was measured at 450 nm to determine the total NAD^+^ concentration. The total NAD^+^ level was normalized to the total protein concentration.

The ATP concentration was quantified using an Enhanced ATP Assay Kit (Beyotime, S0027). Cell lysates were rapidly mixed with the ATP detection working solution, and chemiluminescence was measured using a luminometric plate reader (Promega Biotech Co. Ltd., China).

### 4.19 Caspase‐3 Activity Assay

Caspase‐3 activity was measured using the Caspase‐3 Activity Assay Kit (Beyotime, C1115) according to the manufacturer's instructions. Briefly, cells were lysed with an appropriate amount of lysis buffer. The resulting supernatant was collected after centrifugation at 16 000 *×g* for 15 min at 4°C. A specified amount of the supernatant was mixed with the assay buffer and Ac‐DEVD‐*p*NA, followed by incubation at 37°C for 2 h. Subsequently, the absorbance was measured at 405 nm.

### 4.20 Immunofluorescence Staining

For immunofluorescence staining, processed cells or 4 µm‐thick longitudinal bone sections were first permeabilized with 0.1% Triton X‐100. Subsequently, samples were blocked with 5% bovine serum albumin at RT for 1 h. Thereafter, samples were incubated with corresponding primary antibodies at 4°C overnight. After thorough washing with PBS, the samples were incubated with fluorophore‐conjugated secondary antibodies at RT for 1 h. Nuclei were stained with DAPI for 2 min. Finally, slides were mounted using anti‐fade ProLong Gold medium, then sealed with nail polish prior to imaging. All images were captured using a confocal laser scanning microscope (LSM 900, Zeiss, Germany). For detailed information on all relevant antibodies, please refer to Table S1.

### 4.21 Western Blot Analysis

Total protein from mouse femora was extracted using the Minute Total Protein Extraction Kit for Bone Tissue (Invent, USA) according to the manufacturer's instructions. For cellular protein extraction, cells were lysed with RIPA buffer supplemented with PMSF, protease inhibitor cocktail, and phosphatase inhibitor cocktail (Epizyme, China). Protein concentration was determined with a BCA assay kit (Beyotime, P0009). A total of 40 µg of total protein was loaded onto SDS‐PAGE gels (Epizyme, China), followed by electrophoretic transfer onto PVDF membranes (0.22/0.45 µm, Millipore). Following blocking with 5% non‐fat milk at RT for 1 h, the membranes were then incubated with corresponding primary antibodies at 4°C for 16–18 h. After washing, membranes were incubated with horseradish peroxidase‐conjugated secondary antibodies at RT for 1 h. Antibody‐antigen complexes were visualized using an enhanced chemiluminescence reagent (Millipore, WBLUM0100) and captured with a multi‐color fluorescence imaging system (Amersham Imager 600, GE, USA). To assess loading controls, bound antibodies were removed from the membranes using stripping buffer (Epizyme, PS107). The membranes were re‐blocked with 5% non‐fat milk for 1 h at room temperature and subsequently incubated with an anti‐beta‐actin antibody (Proteintech, HRP‐66009) at 4°C for 4 h. Band densities were quantified using ImageJ. For detailed information on all relevant antibodies, please refer to Table S1.

### 4.22 RT‐qPCR Analysis

Total RNA was extracted from cells using TRNzol Universal (TIANGEN, DP424) and reverse‐transcribed into cDNA using the PrimeScript RT Master Mix (TaKaRa, RR036A). Subsequent qPCR was performed on a LightCycler 96 system using TB Green Premix Ex Taq (TaKaRa, RR820A). For comprehensive primer sequence details, please refer to Table S2.

### 4.23 Statistical and Reproducibility

Data are presented as mean ± SD, with sample sizes as indicated in figure legends. All measurements were obtained from independent samples. Each biological replicate was measured in technical duplicates, and the mean of technical replicates was used as a single data point for statistical analysis. Regarding in vivo experiments, 6–9 mice per group were used, with randomization and blinding applied. In vitro experiments were performed with a minimum of three independent replicates and without data exclusion. All analyses were performed using GraphPad Prism 8. Shapiro‐Wilk normality test was performed for data distribution. Comparisons between two groups were performed using 2‐tailed Student's t‐test, Mann‐Whitney U, or Fisher's exact test, as appropriate. Differences among groups were evaluated using one‐way or two‐way ANOVA, followed by Dunnett's multiple comparisons test or Tukey's test for post‐hoc comparisons. A *p*‐value  <  0.05 was considered statistically significant.

## Author Contributions

Conceptualization, Z.C.L., N.H.L., Y.J.W. and W.X.C., Methodology, Z.C.L., K.H.G., M.J.W., S.L.L., D.D.L., and G.Q.T., Investigation, H.P.X., Validation and Data Curation, Z.C.L., Y.J.L, P.Z., and Z.W.X., Resources, Y.J.W., Writing – Original Draft, Z.C.L., K.H.G. and M.J.W.; Writing – Review & Editing, Z.C.L., N.H.L.; Supervision, W.X.C. and Z.W.X.; Funding Acquisition, N.H.L., Y.J.W., W.X.C., Z.W.X. and P.Z.

## Conflicts of Interest

The authors declare no conflicts of interest.

## Supporting information




**Supporting File**: advs77571‐sup‐0001‐SuppMat.doc.

## Data Availability

The data that support the findings of this study are available from the corresponding author upon reasonable request.
