# Supplementary material for: Bone Marrow Macrophage STING Signaling is a Direct Nexus Linking Marrow Adipose Expansion to NAD+ Depletion and Osteogenic Decline in Skeletal Aging
Source: Adv Sci (Weinh). 2026 Oct 15:e77571. Online ahead of print. doi: 10.1002/advs.77571 (PMC13669683; doi:10.1002/advs.77571)
Supplement: Supplementary file 1 [file ADVS-9999-e77571-s001.doc]

**SUPPLEMENTARY MATERIALS**

**Bone marrow macrophage STING signaling is a direct nexus linking marrow adipose expansion to NAD^+^ depletion and osteogenic decline in skeletal aging**

Zhichao Li^1,2,3^, Kuanhui Gao^2^, Mengjie Wang^3^, Songlin Liang^2^, Dandan Li^4^, Guoqing Tan^3^, Haipeng Xue^3^, Yujie liu^1^, Peng Zhang^1,5,6^ , Zhanwang Xu^2,3^, Wenxiang Cheng^1^*, Yongjun Wang^7,8,9^*, Nianhu Li^2,3^*

^1^ Center for Translational Medicine Research and Development, Shenzhen Institute of Advanced Technology, Chinese Academy of Sciences, Shenzhen, 518055, China;

^2^ First College of Clinical Medicine, Shandong University of Traditional Chinese Medicine, Jinan, 250014, China;

^3^ Department of Orthopedics, Affiliated Hospital of Shandong University of Traditional Chinese Medicine, Jinan, 250014, China;

^4^ College of Integrated Traditional Chinese and Western Medicine, Hebei University of Chinese Medicine, Shijiazhuang, 050011, China;

^5^ Faculty of Biomedical Engineering, Shenzhen University of Advanced Technology, Shenzhen, 518055, China;

^6^ Key Laboratory of Biomedical Imaging Science and System, Chinese Academy of Sciences, Shenzhen, 518000, China;

^7^ Longhua Hospital, Shanghai University of Traditional Chinese Medicine, 200032, Shanghai, China;

^8^ Spine Institute, Shanghai University of Traditional Chinese Medicine, 200032, Shanghai, China;

^9^ Key Laboratory of Theory and Therapy of Muscles and Bones, Ministry of Education (Shanghai University of Traditional Chinese Medicine), 201203, Shanghai, China.

* Correspondence to:

Nianhu Li, MD, PhD

First College of Clinical Medicine, Shandong University of Traditional Chinese Medicine

Jinan, Shandong, CHINA 250014

E-mail address: [tigerlee073@126.com](mailto:tigerlee073@126.com)

Yongjun Wang, MD, PhD

Longhua Hospital, Shanghai University of Traditional Chinese Medicine

Shanghai, CHINA 200032

E-mail address: wangyongjun@shutcm.edu.cn.

Wenxiang Cheng, PhD

Center for Translational Medicine Research and Development, Shenzhen Institute of Advanced Technology, Chinese Academy of Sciences

Shenzhen, Guangdong, CHINA 518055

E-mail address: [wx.cheng@siat.ac.cn](mailto:wx.cheng@siat.ac.cn)

**This supplementary file includes:**

Supplementary Figures 1 to 11

Supplementary Tables 1 to 2

**Supplementary Figure 1**

**Fig. S1** **a** Quantification of western blotting for cGAS, STING, TBK1, IRF3, NF-κB, and IκB protein/phosphorylation levels in bone marrow from 3-, 10-, 18-, and 24-month-old male mice (n = 3 biological replicates). **b** Quantification of the percentage of p-STING and F4/80 double-positive cells in BMMs from 3- and 24-month-old male mice (n = 6 biological replicates). Data are means ± SD.

**Supplementary Figure 2**

**Fig. S2** **a** Representative immunoblots of STING protein level in isolated BMMs of WT and *Sting^ΔMφ^* mice. **b** Body weight of 10- and 18-month-old WT and *Sting^ΔMφ^* mice (n = 15 biological replicates). **c** Quantitative analysis of BMD from 3-, 10-, 18-, and 24-month-old male WT and *Sting^ΔMφ^* mice (n = 9 for 3-month-old WT mice/*Sting^ΔMφ^* mice; n = 7 for 10-month-old WT mice/*Sting^ΔMφ^* mice; n = 8 for 18-month-old WT mice/*Sting^ΔMφ^* mice; n = 7 for 24-month-old WT mice/*Sting^ΔMφ^* mice). **d** Quantitative analysis of the mean differences in BV/TV, Tb.N, Tb.Th, and Tb.Sp between WT and *Sting^ΔMφ^* mice at 10 and 18 months of age (n = 7 10-month-old WT mice; *Sting^ΔMφ^* mice, n = 8 18-month-old WT mice; *Sting^ΔMφ^* mice). **e** Quantitative analysis of adipocyte number in 3-, 10-, 18-, and 24-month-old WT and *Sting^ΔMφ^* mice (n = 6 biological replicates). **f** Quantitative analysis of MAR and MS/BS in 3-, 10-, 18-, and 24-month-old WT and *Sting^ΔMφ^* mice (n = 6 biological replicates). **g** Representative immunoblots of STING protein level in isolated BMMs of AAV-BMM-shNC-treated or AAV-BMM-sh*Sting*-treated mice. **h** Quantitative analysis of BMD from AAV9-BMM-shNC-treated or AAV9-BMM-sh*Sting*-treated 18-month-old mice (n = 6 biological replicates). **i** Quantitative analysis of adipocyte number in AAV-BMM-shNC-treated or AAV-BMM-sh*Sting*-treated 18-month-old mice (n = 6 biological replicates). **j** Quantitative analysis of MAR and MS/BS in AAV-BMM-shNC-treated or AAV-BMM-sh*Sting*-treated 18-month-old mice (n = 6 biological replicates). Data are means ± SD.

**Supplementary Figure 3**

**Fig. S3** **a** Representative images of Alizarin Red (red) and ALP (purple) staining for osteogenic differentiation activity in BMSCs co-cultured with BMMs from 3- or 18-month-old WT and 18-month-old *Sting^ΔMφ^* mice. **b** Representative images of Calcein-AM-based tube formation assay in bEnd.3 cells co-cultured with BMMs from 3- or 18-month-old WT and 18-month-old *Sting^ΔMφ^*, and corresponding quantification (n = 6 biological replicates). **c** Quantification of western blotting for cGAS, STING, TBK1, IRF3, NF-κB, and IκB protein/phosphorylation levels in BMMs from PBS-treated or LPS-treated male mice (n = 3). **d** Quantification of western blotting for cGAS, STING, TBK1, IRF3, NF-κB, and IκB protein/phosphorylation levels in BMMs from PBS-treated or 3 M-/18 M-SN-treated male mice (n = 3 biological replicates). **e** Quantification of western blotting for cGAS, STING, TBK1, IRF3, NF-κB, and IκB protein/phosphorylation levels in BMMs from PBS-treated or 3 M-/18 M-BMAds-CM-treated male mice (n = 3 biological replicates). **f** Quantification of western blotting for cGAS, STING, TBK1, IRF3, NF-κB, and IκB protein/phosphorylation levels in BMMs co-cultured with 3 M-/18 M-BMAds (n = 3 biological replicates). Data are means ± SD.

**Supplementary Figure 4**

**Fig. S4** **a** Quantitative analysis of BMD from PBS-treated or rDPP4-treated male mice (n = 6 biological replicates). **b** Representative immunoblots of DPP4 protein level in isolated BMAds of AAV-BMAd-shNC-treated or AAV-BMAd-sh*Dpp4*-treated mice. **c** Quantitative analysis of BMD from AAV9-BMAd-shNC-treated or AAV9-BMAd-sh*Dpp4*-treated male mice (n = 6 biological replicates). **d** Quantification of western blotting for cGAS, STING, TBK1, IRF3, NF-κB, and IκB protein/phosphorylation levels in BMMs from PBS-treated or rDPP4-treated male mice (n = 3 biological replicates). **e** Endotoxin levels in femoral bone marrow from PBS-treated or rDPP4-treated male mice (n = 6 biological replicates). **f** Quantification of western blotting for cGAS and STING protein/phosphorylation levels in BMMs treated with either rDPP4 alone or in combination with 18 M-SN (n = 3 biological replicates). Data are means ± SD.

**Supplementary Figure 5**

**Fig. S5** **a** Representative immunoblots of STING protein/phosphorylation level in BMMs and quantification analysis. BMMs were either pretreated with Ad-sh*Cav-1* or treated in the presence of GB83 or M6P, followed by stimulation with SN-rDPP4 (n = 3 biological replicates). **b** Quantification of western blotting for TLR2 and TLR4 protein levels in BMMs from rDPP4-treated or SN-rDPP4-treated male mice (n = 3 biological replicates). **c** Quantification of western blotting for TAK1 and STING protein/phosphorylation levels in BMMs stimulated with SN-rDPP4 and treated with C29 or Resatorvid (n = 3 biological replicates). **d** Representative immunoblot of STING protein/phosphorylation level in BMMs from PBS-treated or resatorvid-treated male mice (n = 3 biological replicates). **e** Quantification of western blotting for cGAS, STING, TBK1, IRF3, NF-κB, and IκB protein/phosphorylation levels in BMMs treated with EtBr (n = 3 biological replicates). **f** Representative immunoblots of STING and cGAS protein levels in BMMs knocked down with Ad-sh*Sting* or Ad-sh*Cgas*. Data are means ± SD.

**Supplementary Figure 6**

**Fig. S6** **a** Schematic of key enzymes in NAD^+^ biosynthesis pathways. **b** qPCR analysis of key enzymes in the NAD^+^ de novo biosynthesis pathway in 3 M-, 10 M-, 18 M-, 24 M-BMMs (n = 6 biological replicates). **c** Quantification of western blotting for key enzymes in the NAD^+^ salvage biosynthesis pathway (n = 3 biological replicates). **d** WST-8 analysis of NAD^+^ levels in 3 M-BMMs, 18 M-BMMs or 18 M-BMMs treated with 78c (n = 6 biological replicates). **e - g** WST-8 analysis and fluorometric assay of total NAD^+^ levels (**e, f**) and CD38 NADase activity (**g**) in BMMs with or without *Cgas*, *Sting*, *Irf3*, and *Rela* knockdown, in both diABZI-treated and co-cultured with 18 M-BMAds (n = 6 biological replicates). **h, i** qPCR (n = 6 biological replicates) and western blotting (n = 3 biological replicates) analysis of *Cd38* mRNA level (**h**) and CD38 protein (**i**) in BMMs with or without *Cgas*, *Sting*, *Irf3*, and *Rela* knockdown, in both diABZI-treated, SN-rDPP4-treated and co-cultured with 18 M-BMAds. **j** Representative immunoblots of IRF3 or NF-κB P65 protein levels in BMMs knocked down with Ad-shIrf3 or Ad-shRela. **k** Identification of putative IRF3 and P65 binding sites upstream of the 5'-untranslated region of the CD38 gene. Data are means ± SD.

**Supplementary Figure 7**

**Fig. S7** **a** qPCR analysis of *Nos2*, *Tnfα*, *Il1b*, and *Il6* mRNA levels in WT-3-/WT-18-BMMs and *Sting^ΔMφ^*-18 M-BMMs (n = 6 biological replicates). **b** qPCR analysis of *Tnfα*, *Il1b*, and *Il6* mRNA levels in BMMs with or without *Cgas* and *Sting* knockdown, in both SN-rDPP4-treated and co-cultured with 18 M-BMAds (n = 6 biological replicates). **c** qPCR analysis of *Tnfα*, *Il1b*, and *Il6* mRNA levels in BMMs **following co-treatment with 78c and SN-rDPP4, and in BMMs co-cultured with 18 M-BMAds with 78c (n = 6** biological replicates**). d** Representative immunoblots of CD38 protein level in isolated BMMs of AAV-BMM-shNC-treated or AAV-BMM-sh*Cd38*-treated mice. **e** Quantitative analysis of BMD from AAV9-BMM-shNC-treated or AAV9-BMM-sh*Cd38*-treated male mice (n = 6 biological replicates). **f** qPCR analysis of *Cdkn2a*, *Cdkn1a*, *Nos2*, *Tnfα*, *Il1b*, *Il6*, *Ym1*, *Fizz1*, and *Arg1* mRNA levels in bone marrow from AAV-BMM-shNC-treated or AAV-BMM-sh*Cd38*-treated mice (n = 6 biological replicates). **g** WST-8 analysis of NAD^+^ levels in bone marrow from AAV-BMM-shNC-treated or AAV-BMM-sh*Cd38*-treated mice (n = 6 biological replicates). **h** Representative immunoblots of Slc12a8 protein level in BMSCs. Data are means ± SD.

**Supplementary Figure 8**

**Fig. S8** **a** Schematic of the co-culture system related to data shown in (**b**). **b** WST-8 analysis of NAD^+^ levels in 18 M-BMSCs treated with NMN and co-cultured with 3M-BMMs that were first subjected to *Cgas* or *Sting* knockdown and then to either co-culture with 18 M-BMAds or SN-rDPP4 treatment (n = 6 biological replicates). **c** Schematic of the co-culture system related to data shown in (**d**). **d** WST-8 analysis of NAD^+^ levels in 18 M-BMSCs co-cultured with BMMs from 18-month-old WT or *Sting^ΔMφ^* mice and treated with NMN (n = 6 biological replicates). **e** WST-8 analysis of NAD^+^ levels in bone marrow from 3- or 18-month-old WT and 18-month-old *Sting^ΔMφ^* mice (n = 6 biological replicates). **f** Schematic of the co-culture system related to data shown in (**g**). **g** WST-8 analysis of NAD^+^ levels in BMSCs from 18-month-old *Sting^ΔMφ^* mice with BMM-specific overexpression of either WT CD38 or Δ49 CD38 (n = 6 biological replicates). Data are means ± SD.

**Supplementary Figure 9**

**Fig. S9** **a - c** Representative TMRE staining (**a**), ATP concentration (**b**) and caspase-3 activity (**c**) analysis in 3 M-BMSCs, 18 M-BMSCs or 18 M-BMSCs treated with NMN and/or co-cultured with 3 M-BMMs (scale bar: 50 μm; n = 6 biological replicates). **d - f** Representative TMRE staining (**d**), ATP concentration (**e**) and caspase-3 activity (**f**) analysis in 3 M-BMSCs treated with NMN and co-cultured with 3 M-BMMs, 18 M-BMMs, diABZI-treated 3 M-BMMs, SN-rDPP4-treated 3 M-BMMs, or 3 M-BMMs co-cultured with 18 M-BMAds, respectively (scale bar: 50 μm; n = 6 biological replicates). **g - i** Representative TMRE staining (**g**), ATP concentration (**h**) and caspase-3 activity (**i**) analysis in 18 M-BMSCs treated with NMN and co-cultured with *Cgas*- or *Sting*-knockdown 3 M-BMMs that were treated with diABZI (scale bar: 50 μm; n = 6 biological replicates). **j - l** Representative TMRE staining (**j**), ATP concentration (**k**) and caspase-3 activity (**l**) analysis in 18 M-BMSCs treated with NMN and co-cultured with *Cgas*- or *Sting*-knockdown 3 M-BMMs that were treated with SN-rDPP4 (scale bar: 50 μm; n = 6 biological replicates). **m - o** Representative TMRE staining (**m**), ATP concentration (**n**) and caspase-3 activity (**o**) analysis in 18 M-BMSCs treated with NMN and co-cultured with *Cgas*- or *Sting*-knockdown 3 M-BMMs (pre-co-cultured with 18 M-BMAds) (scale bar: 50 μm; n = 6 biological replicates). Data are means ± SD.

**Supplementary Figure 10**

**Fig. S10** **a**, **b** ATP concentration (**a**) and Caspase-3 activity (**b**) analysis in WT-3 M-/WT-18 M-BMSCs and *Sting^ΔMφ^*-18 M-BMSCs (n = 6 biological replicates). **c** Quantification of western blotting for RUNX2 and BMP2 protein levels in WT-3 M-/WT-18 M-BMSCs and *Sting^ΔMφ^*-18 M-BMSCs (n = 3 biological replicates). **d**, **e** ATP concentration (**d**) and Caspase-3 activity (**e**) analysis in BMSCs from 3- or 18-month-old AAV-BMM-shNC-treated or 18-month-old AAV-BMM-sh*Cd38*-treated mice (n = 6 biological replicates). **f** Quantification of western blotting for RUNX2 and BMP2 protein levels in BMSCs from 3- or 18-month-old AAV-BMM-shNC-treated or 18-month-old AAV-BMM-sh*Cd38*-treated mice (n = 3 biological replicates). Data are means ± SD.

**Supplementary Figure 11**

**Fig. S11** **a** Quantitative analysis of BMD and Tb.Th from PBS-treated, ALD-treated, or H-151 and NMN co-treated mice (n = 6 biological replicates). **b** Representative images of calcein double labeling (scale bar: 50 μm) and quantitative analysis of MAR (n = 6 biological replicates). **c** qPCR analysis of *Cd38*, *Cdkn2a*, *Cdkn1a*, *Nos2*, *Tnfα*, *Il1b*, *Il6*, *Ym1*, *Fizz1*, and *Arg1* mRNA levels in bone marrow from 3-month-old, 18-month-old or H-151 and NMN co-treated 18-month-old mice (n = 6 biological replicates). **d** Representative images of TMRE staining of mitochondrial membrane potential in BMSCs from 3-month-old, 18-month-old or H-151 and NMN co-treated 18-month-old mice (scale bar: 50 μm). **e** Representative images of Calcein-AM (green) and mito tracker (red) staining for mitochondrial permeability transition pore opening in BMSCs from 3-month-old, 18-month-old or H-151 and NMN co-treated 18-month-old mice (scale bar: 50 μm). **f** OCR analysis in BMSCs from 3-month-old, 18-month-old or H-151 and NMN co-treated 18-month-old mice, with quantitative analysis of basal and maximal respiration levels (n = 6 biological replicates). **g**, **h** ATP concentration (**g**) and caspase-3 activity (**h**) analysis in BMSCs from 3-month-old, 18-month-old or H-151 and NMN co-treated 18-month-old mice (n = 6 biological replicates). **i** WST-8 analysis of NAD^+^ levels in BMSCs from 3-month-old, 18-month-old or H-151 and NMN co-treated 18-month-old mice (n = 6 biological replicates). Data are shown as mean ± SD.

**Supplementary Table 1**

**Table. S1:** Antibodies catalogue

| **Antibody name** | **Source** | **Identifier** |
| --- | --- | --- |
| Anti-cGAS | Abcam | Cat# ab252416 |
| Anti-STING | Abcam | Cat# ab288157 |
| Anti-Phospho-STING | Cell Signaling Technology | Cat# 72971 |
| Anti-Phospho-STING | Cell Signaling Technology | Cat# 62912 |
| Anti-TBK1 | Abcam | Cat# ab40676 |
| Anti-Phospho-TBK1 | Cell Signaling Technology | Cat# 5483 |
| Anti-IRF3 | Abcam | Cat# ab68481 |
| Anti-Phospho-IRF3 | Cell Signaling Technology | Cat# 4947 |
| Anti-NF-kB p65 | Abcam | Cat# ab16502 |
| Anti-Phospho-NF-kB p65 | Abcam | Cat# ab76302 |
| Anti-IκBα | Abcam | Cat# ab32518 |
| Anti-Phospho-IκBα | Cell Signaling Technology | Cat# 2859 |
| Anti-Perilipin-1 | Cell Signaling Technology | Cat# 9349 |
| Anti-P21 | Abcam | Cat# ab109199 |
| Anti-P16 | Abcam | Cat# ab211542 |
| Anti-DPP4 | Abcam | Cat# ab222716 |
| Anti-TAK1 | Proteintech | Cat# 12330-2-AP |
| Anti-TLR4 | Proteintech | Cat# 66350-1-Ig |
| Anti-TLR2 | Cell Signaling Technology | Cat# 13744 |
| Anti-Phospho-TAK1 | Cell Signaling Technology | Cat# 4508 |
| Anti-DYKDDDDK | Cell Signaling Technology | Cat# 14793 |
| Anti-HA | Cell Signaling Technology | Cat# 3724 |
| Anti-HSP60 | Abcam | Cat# ab190828 |
| Anti-dsDNA | Abcam | Cat# ab27156 |
| Anti-CD38 | Thermo Fisher | Cat# PA5-89326 |
| Anti-SIRT1 | Abcam | Cat# ab11034 |
| Anti-SIRT3 | Abcam | Cat# ab246522 |
| Anti-SIRT5 | Abcam | Cat# ab259967 |
| Anti-SIRT7 | Abcam | Cat# ab259968 |
| Anti-PARP1 | Abcam | Cat# ab191217 |
| Anti-NAMPT | Proteintech | Cat# 11776-1-AP |
| Anti-NMNAT1 | Proteintech | Cat# 28493-1-AP |
| Anti-SLC12A8 | Proteintech | Cat# 12242-1-AP |
| Anti-RUNX2 | Abcam | Cat# ab192256 |
| Anti-BMP2 | Abcam | Cat# ab214821 |
| Anti-Beta Actin | Proteintech | Cat# HRP-66009 |

**Supplementary Table 2**

**Table. S2: Primers used for RT-qPCR analysis**

| **Gene name** | **Primer direction** | **Primer sequence** |
| --- | --- | --- |
| *Cgas* | FORWARD | GAGGCGCGGAAAGTCGTAA |
|  | REVERSE | TTGTCCGGTTCCTTCCTGGA |
| *Sting* | FORWARD | GGCTGGCCTGGTCATACTAC |
|  | REVERSE | GCACCACTGAGCATGTTGTT |
| *Cd38* | FORWARD | TCTCTAGGAAAGCCCAGATCG |
|  | REVERSE | GTCCACACCAGGAGTGAGC |
| *Nampt* | FORWARD | TGGGGTGAAGACCTGAGACA |
|  | REVERSE | TCCACGCCATCTCCTTGAAT |
| *Nmnat1* | FORWARD | GCTGTGTGGGGCAGATTTACT |
|  | REVERSE | CCCGAGTGATACAGATGAGCC |
| *Nmnat2* | FORWARD | AGAACACCCAGCCCATTTAC |
|  | REVERSE | GAGGCTTTCTCCCACCTTTC |
| *Nmnat3* | FORWARD | TCCTGGGGAGTAGGGGTATTC |
|  | REVERSE | AGCAGGTCTCCTCCTTAGTGA |
| *Sirt1* | FORWARD | TATCTATGCTCGCCTTGCGG |
|  | REVERSE | GACACAGAGACGGCTGGAAC |
| *Sirt2* | FORWARD | GGCAGTTCAAGCCAACCATC |
|  | REVERSE | CTCTTTCATCCAGCCCATCG |
| *Sirt3* | FORWARD | GCTTCTGCGGCTCTATACACA |
|  | REVERSE | GCTGTTACAAAGGTCCCGTG |
| *Sirt4* | FORWARD | GGACACGGTGAACCCAGACAAG |
|  | REVERSE | GCGGGCGGTGAGGATGAAC |
| *Sirt5* | FORWARD | AGAGACTCAAGACGCCAGAATCC |
|  | REVERSE | AGCAAGCCTCCGCATCCTG |
| *Sirt6* | FORWARD | TTTCGTATAAGTCCAAGCCCAACAG |
|  | REVERSE | GGCAGCCTCGGTCTTCACTC |
| *Sirt7* | FORWARD | AAGCGAAGCGGAGCCTACC |
|  | REVERSE | CCATCACAGTTCTGAGACACCAC |
| *parp1* | FORWARD | CTGATGTTGAGGTGGATGGCT |
|  | REVERSE | AAGTCACCCAATGTCTTCTCCG |
| *parp2* | FORWARD | TGCTAGAGGCATTGGGAGAC |
|  | REVERSE | ACGCAAAGCACAGTGTAGGT |
| *Afmid* | FORWARD | CGTGGTGGTGATAGTGGCTTATG |
|  | REVERSE | AGAGTGTCCGCAGAGGTAGATTC |
| *kyat1* | FORWARD | GTCCAAGGAGTATGATGTCGTGAAC |
|  | REVERSE | ACGCTGAGGTGTACTGGTTGAG |
| *Kmo* | FORWARD | ACAGTGCTCGGACCTGACAAG |
|  | REVERSE | CTCTGACAGTTGAATAGGCTCCATC |
| *Qprt* | FORWARD | ATGCCACCGCTACGACCTG |
|  | REVERSE | TGCTACATTCCACCTCTACCTTCAG |
| *Haao* | FORWARD | CTGGAGAGCGAGCTGGATGG |
|  | REVERSE | GTGCTAACTGCGTGCCAAGG |
| *kynu* | FORWARD | CACGGACTTGATGTTGAGAA |
|  | REVERSE | TTTGTTATGGCAGGAATGTTG |
| *ido1* | FORWARD | GAACCGAGGGGATGACGATG |
|  | REVERSE | AGTGGGGATTGCTTTGATTGC |
